# FARESHARE: An open-source apparatus for assessing drinking microstructure in socially housed rats

**DOI:** 10.1038/s44277-024-00002-z

**Published:** 2024-02-27

**Authors:** Jude A. Frie, Jibran Y. Khokhar

**Affiliations:** 1 Department of Biomedical Sciences, University of Guelph, Guelph, ON Canada; 2 Department of Anatomy and Cell Biology, University of Western Ontario, London, ON Canada

**Keywords:** Reward, Addiction

## Abstract

Social factors have been shown to play a significant and lasting role in alcohol consumption. Studying the role of social context on alcohol drinking is important to understand the factors that contribute to the initiation or maintenance of casual and problematic alcohol use, as well as those that may be protective. A substantial body of preclinical research has shown that social environment such as housing conditions and social rank plays an important role in alcohol consumption and preference, though the extent of these effects have been obfuscated by methodological differences and technical challenges. Robust individual differences in alcohol intake in socially housed animals are difficult to track when animals share a common fluid source. Commercial solutions are prohibitively expensive and are limited by proprietary software and hardware (including caging systems). Here we describe FARESHARE, an affordable, open-source solution for tracking fluid consumption in socially housed rats. The device uses RFID and custom hardware to individually measure and record each rat’s fluid consumption and licking microstructure. Each bout is also timestamped such that the circadian effects of drinking behaviour may be analysed. We provide a validation showing the operation of the device in a two-bottle-choice alcohol-drinking paradigm over a nine-day period in four group-housed female rats. We show that FARESHARE is able to capture traditional measures such as daily intake and preference, as well as circadian effects, microstructure, and individual variations in drinking.

## Lay summary

This study introduces FARESHARE, an affordable and open-source solution for tracking fluid consumption in socially housed rats to better understand the influence of social housing on alcohol consumption. The device uses wireless technology and custom hardware to measure and record each rat’s fluid intake and licking patterns, addressing challenges in accurately assessing individual differences in alcohol intake. The researchers validated FARESHARE, demonstrating its ability to capture circadian effects, microstructure, and individual variations in drinking behavior.

## Introduction

Given the lack of new effective therapeutics in addiction treatment despite a substantial and rich mechanistic preclinical literature, there has been a recent effort to improve the translational validity of animal models by increasing the ecological relevance of experiments [1]. Previous findings have shown that the risk of developing substance use, and the effectiveness of treatment interventions depend greatly on the social environment [2]. The role of social factors such as peer pressure, loneliness, and social stress and support on alcohol use has been a large focus in clinical research, with significant facilitation of binge drinking by peers, increased risk of alcohol problems in solitary drinkers, and gender differences in motives for alcohol consumption in social settings [3–5]. Therefore, addressing the lack of preclinical models incorporating social context and behaviour has been highlighted as a major opportunity for improving translation in animal models of alcohol use disorder [6–8].

Preclinical studies have shown that the presence of other animals may facilitate or reduce alcohol drinking depending on the particular social environment such as housing conditions and social rank [9]. Additionally, individual variation in juvenile social play has been shown to predict future alcohol intake and loss of control over alcohol seeking in rats, highlighting social behaviour as a determinant of alcohol-directed behaviour [10]. Many outstanding questions remain concerning social context and behaviour in alcohol use but are difficult to study due to methodological inconsistencies and technical challenges. The role of social rank on drinking behaviour obfuscates any findings tracking group consumption, therefore necessitating individual tracking to fully characterize social drinking behaviour. However, it is difficult to track individual differences in consumption when animals share a cage and a common substance source. The majority of drug intake studies in rodents have required social isolation of subjects to get around this issue, neglecting the social context so relevant to clinical populations [2, 11]. Radio frequency tracking is one promising solution and has been used in commercial products such as IntelliCage. IntelliCage, however, is prohibitively expensive (~$65,000 USD per cage) and as a commercial product is limited by proprietary software and hardware, including the incompatibility with existing caging systems. Additionally, individual drinking is measured with only a lickometer, and while licking has been shown to be correlational with the volume of consumption, the number of licks in any given bout may vary with the drinking context [12].

It is clear that there is a need for a non-commercial, open-source alternative to improve the quality and availability of preclinical alcohol research in social settings. A general use, low-cost method for measuring individual momentary fluid intake in socially housed rats is warranted, especially as all open-source methods for social fluid tracking to date have been developed for mice [13–15]. Though mice are the more popular model for many areas of research, rat models are still of unique importance to alcohol research. Indeed, there are rat models that have yet to be demonstrated in mice such as compulsive drinking in a relapse situation [16]. Thus, we developed a device for **F**luid **A**cquisition **RE**cording in **S**ocially **H**oused **A**nimal **RE**search (FARESHARE) to allow for RFID-based fluid tracking in rats.

FARESHARE is an affordable, open-source device that can be placed in any home cage or operant box that can be used with as many animals as required for the study design. It uses short-range RFID to identify individual rats, a lickometer to determine when animals are drinking to begin fluid delivery, custom low-profile PCB that sits directly on top of an Arduino-based microcontroller, volumetric fluid delivery via a custom peristaltic pump for accurate measurement of consumption volume (that may also be used for other purposes), OLED display for showing overall individual fluid consumption and licks, and continuous data logging to an SD card module. Here we validate FARESHARE via a two-bottle choice paradigm. Having a robust, affordable method for measuring drinking microstructure in socially housed animals will be of considerable benefit in preclinical addiction research, and a step toward more translationally relevant animal models of fluid consumption. The added dimension of time allows for the analysis of circadian-linked consumption and the discrimination of binge-like drinking behaviours. Additionally, the open-source nature of the project enables researchers to customize the device for more advanced applications such as sending signals to additional peripherals (e.g., optogenetic stimulation or electrophysiology/fiber photometry) or software on drinking initiation for time-locked or closed-loop interventions, manipulations, and measurements.

## Materials and Methods

### Animals

Four female Sprague Dawley rats (Charles River Lab, St. Constant, Canada) were kept on a 12:12 light-dark cycle (lights off at 0800 h) in a colony maintained at 21 °C in a single polyethylene Guinea pig cage. Food (Envigo, Madison, Wisconsin, USA, Rodent Diet, 14% protein) and water were available ad libitum. All animal procedures were performed in accordance with the University of Guelph Animal Care Committee, and the University of Western Ontario Animal Use Subcommittee, and were consistent with guidelines established by the Canadian Council on Animal Care.

### Injection of RFID tags

Rats were injected subcutaneously with 5 mg/kg carprofen prior to RFID injection. Anesthesia was induced with 4–5% isoflurane. RFID tags were injected using an RFID chip injector under the scalp and RFID was placed such that the tip of the tag sat between the eyes and extended back toward the ears as shown in Supplementary Fig. 1.

### Design of electronics and 3D printer files

FARESHARE uses the Arduino-based RedBoard Qwiic that has a ATmega328 processor with a 16 MHz Clock Speed, 20 I/O pins, and 32 kb of flash memory. All code was written in Arduino language using Arduino IDE with added open-source libraries: Adafruit GFX Library, Adafruit SSD1306, Sparkfun Qwiic OpenLog, and CapacitiveSensor. The PCB was designed in the open-source CAD software Fritzing. The PCB acts as the bridge between the RedBoard, RFID reader (ID-12LA), OLED display, SD card reader, Capacitive lickometer, and motor controller. An A4988 Stepper Motor Driver controls the peristaltic pump’s Nema 17 Stepper Motor. 3D printer files were created in SolidWorks and printed on a Creality K1 Speedy printer using PLA filament with Creality Print slicer.

### Pump characterization

To evaluate the error between volume reading and actual volume, the output tube of one device was placed in a beaker and the beaker was placed on an analytical scale. The device was then activated until 1.00 mL was output on the OLED display and the weight reading on the scale was recorded. The scale was then tared, and another mL was measured. This was repeated 20 times without resetting the FARESHARE to determine if the error would be stable over time.

### Measurement of alcohol consumption and preference

Two reservoirs were prepared in 1L Erlenmeyer flasks (10% ethanol v/v and tap water). Each flask was sealed with parafilm except for a small hole for the FARESHARE input pump hose to go through. Two FARESHARE devices were attached to the inside of one wall of the home cage and primed for either alcohol or water. Rats were then given 24-h access to both devices for nine days.

### Statistical analysis

Statistics were conducted in GraphPad Prism 9.5.1. Pump error was evaluated by simple linear regression and descriptive statistics. Changes in daily alcohol consumption and preference were analyzed via one-way ANOVA with day as a repeated measure. Individual hourly water and alcohol consumption were evaluated via two-way ANOVA with time as a repeated measure. The effect of light cycle and bout range on each subject’s water and alcohol consumption was evaluated via two-way ANOVA. Max and mean bout sizes, volume per lick, and bout frequency between fluid types were analyzed for all nine days via a two-tailed paired *t*-test. Bouts were defined as a drinking event containing at least 20 licks, similar to previous literature [17–20]. A *p* value < 0.05 was considered significant for all comparisons. Results are displayed as mean ± SEM.

## Results

### Hardware and design

**Fig. 1:**
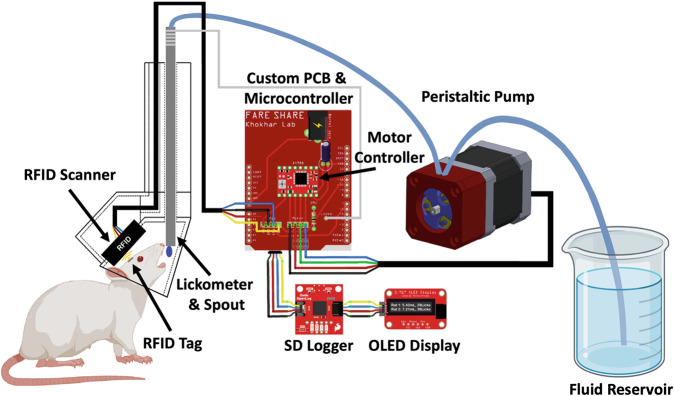
Schematic of FARESHARE. A central Arduino-based microcontroller and PCB are connected to a custom peristaltic pump, SD card reader, OLED display, RFID scanner, and capacitive metal straw that acts as a water spout and lickometer

FARESHARE requires one standard wall receptacle (110 V/60 Hz) for power. A single device can be built for ∼$185 US. The device is low profile requiring little lab space to store or use. FARESHARE uses RFID to identify which animal is drinking. When a rat places its head near the RFID scanner and begins licking, an RFID tag implanted under the rat’s scalp will activate a peristaltic pump sending fluid through a metal straw. The straw is capacitive such that it will measure the number of licks in a drinking bout. The total bout volume and lick number are then logged to an SD card. The total volume delivered and total licks since experiment initiation will also be updated in real-time to an OLED display for real-time updates. The SD card creates a .CSV file with timestamped bouts for each rat. The 3D-printed RFID sensor housing is only large enough for a single rat to fit its head, thus, only one rat is ever measured at a time. This design also uniquely allows rats to take turns drinking while in each other’s presence without the need for a door or separate administration chamber to keep the rat isolated for RFID scanning. The system is based around an Arduino Uno type microcontroller to allow labs to easily customize the device for their specific use case. A custom PCB is included to improve circuit reliability, and to make soldering less intensive. All files can be found on GitHub (https://github.com/jfrie/FARESHARE), including 3D printer files, PCB GERBER files, and Arduino code. A bill of materials, detailed build instructions, and operating instructions are included in supplemental materials. A schematic of the device is shown in Fig. 1.

### Pump characterization

Pump accuracy following 20 measurements of 1.00 mL was high, with an average error of 0.5% (95% CI 0.3–0.7) based on weight. The error also remained stable across tests, with no statistically significant slope following simple linear regression of either raw error (mL; F(1,18) = 0.013, *P* = 0.91) or percent error (F(1,18) = 2.40, *P* = 0.14) as shown in Supplemental Fig. 2. A durability test found the pump was able to run continuously for 30.5 h before signs of tubing failure. As the cumulative alcohol drinking duration of all rats combined over the nine days was 2.2 h, this would correspond to a duration of ~125 days for the current experiment before failure.

### Fluid consumption in group-housed rats

**Fig. 2:**
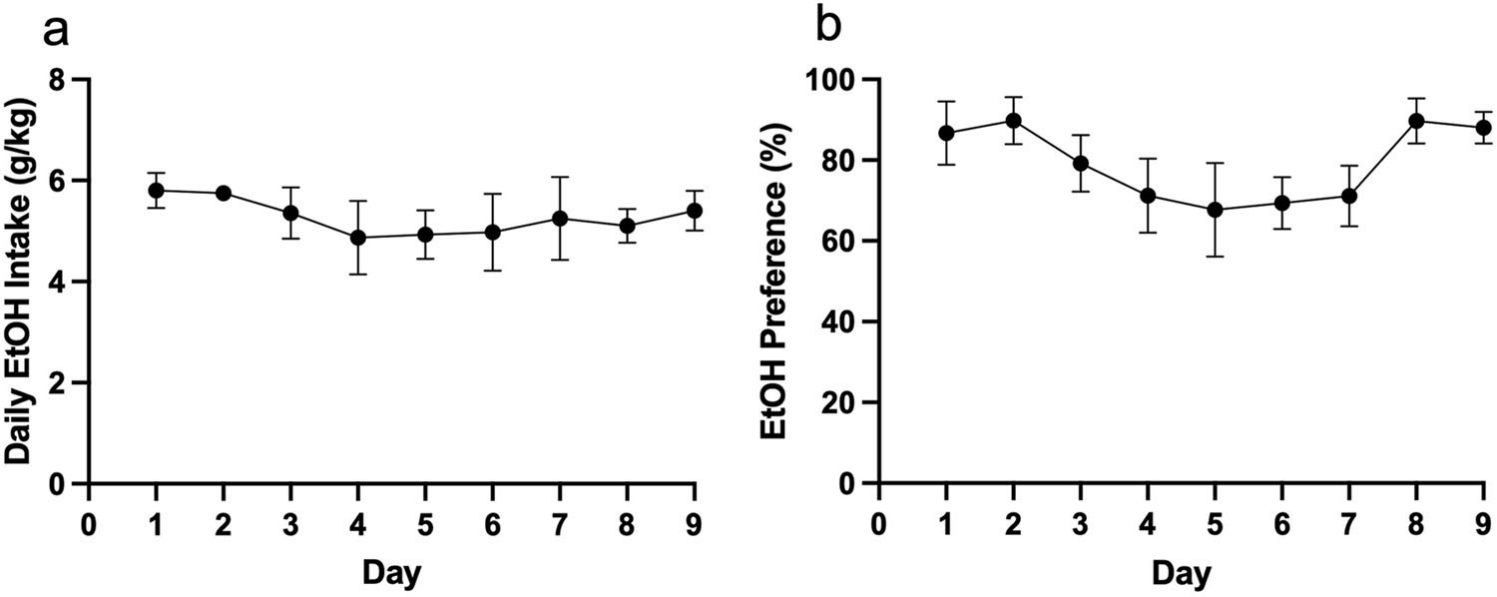
Average alcohol (10% ethanol v/v) consumption in group-housed rats shown as **a** daily intake (g/kg), and **b** preference (%). Daily intake was consistent across days. Preference showed a significant effect of day, indicating shifting preferences across days, though no difference survived post-hoc

Rats showed consistent daily alcohol consumption across days as shown in Fig. 2a. Alcohol preference (Fig. 2b) did shift across days (effect of day, F(1.854,5.561) = 7.455, *P* = 0.028), though no significant differences between days were seen following a Sidak post-hoc test between days.

### Individual fluid consumption

**Fig. 3:**
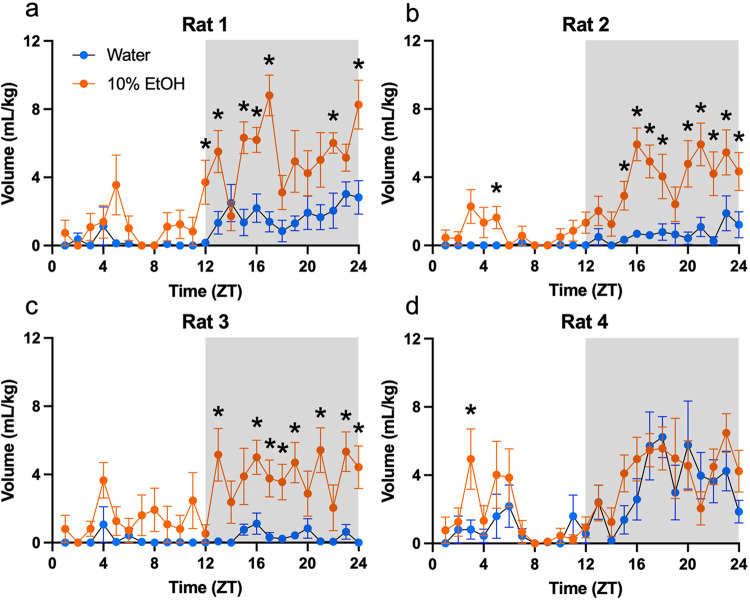
Individual fluid consumption binned by an hour and plotted by Zeitgeber time with grey shading indicating the dark cycle. All rats drank more over time and consumed more alcohol than water. Rats 1–3 (**a**–**c**) also had a time by fluid-type interaction while rat 4 (**d**) did not. *p < 0.05 versus water

Individual fluid intake binned by hour and averaged across all nine days is shown in Fig. 3. All rats showed greater ethanol consumption compared to water (Rat 1: F(1,16) = 90.81, *P* < 0.001; Rat 2: F(1,16) = 137.9, *P* < 0.001; Rat 3: F(1,16) = 161.7, *P* < 0.001; Rat 4: F(1,16) = 4.817, *P* = 0.043) and fluid consumption increased with time (Rat 1: F(6.161,98.57) = 8.151, *P* < 0.001, Rat 2: F(5.713,91.41) = 6.535, *P* < 0.001; Rat 3: F(6.324,101.2) = 2.747, *P* = 0.015; Rat 4: F(23, 368) = 5.620, *P* < 0.001). In 3 of the 4 rats, there was also an interaction effect (Rat 1: F(23,368) = 2.942, *P* < 0.001; Rat 2: F(23,368) = 3.056, *P* < 0.001; Rat 3: F(23,368) = 1.857, *P* = 0.01), with alcohol consumption increasing more than water over time.

### Circadian effects on fluid consumption

**Fig. 4:**
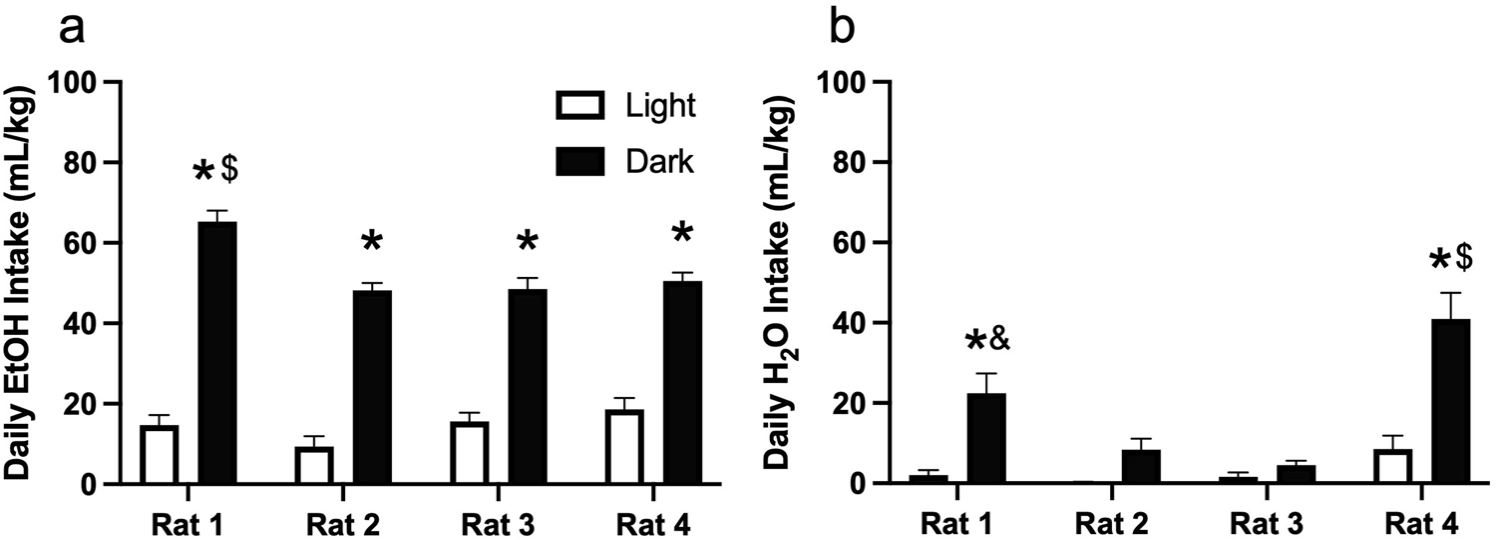
Average daily **a** alcohol and **b** water consumption during the light and dark cycle. Rats consistently drank more during the night cycle. *p < 0.05 dark versus light. $ *p* < 0.05 versus all groups. & *p* < 0.05 vs. rat 2 and 3 dark

Rats consumed a much greater volume of alcohol during the dark cycle (F(1,64) = 494.7, *P* < 0.001) as shown in Fig. 4a. Rats also consumed more water during the dark cycle (F(1,64) = 45.74), *P* < 0.001) as shown in Fig. 4b. Rats showed robust individual differences in fluid consumption of both alcohol (F(3,64) = 7.397, *P* < 0.001) and water (F(3,64) = 17.78, *P* < 0.001).

### Fluid type effects on bout microstructure

**Fig. 5:**
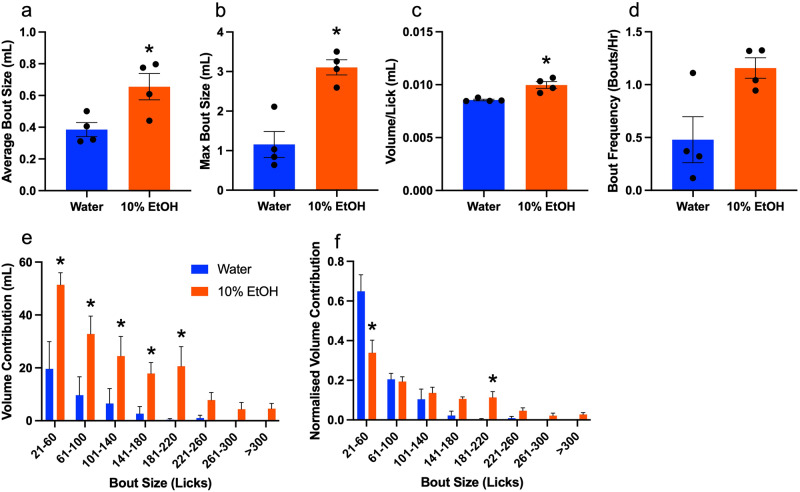
Bout structure analysis across all nine days based on fluid type. Alcohol drinking resulted in greater **a** average bout size, **b** max bout size, and **c** volume per lick. **d** Bout frequency is marginally higher in alcohol compared to water. **e** Overall volume consumed binned by lick number. A greater volume of alcohol was consumed across bout ranges. **f** Volume contributed by each bout range normalized by the total volume of each fluid type. Normalized water consumption is higher than alcohol in small bouts, while normalized alcohol consumption is higher in larger bouts. *p < 0.05 alcohol versus water

Drinking was greatly affected by the fluid type, with the alcohol resulting in greater average bout size (Fig. 5a; t(3) = 3.659, *P* = 0.0353), max bout size (Fig. 5b; t(3) = 7.088, *P* = 0.0058), and volume per lick (Fig. 5c; t(3) = 5.255, *P* = 0.0134). Bout frequency also appeared to be higher but did not reach significance (Fig. 5d; t(3) = 2.455, *P* = 0.0913). The alcohol volume contributed by different bout ranges was also consistently greater than water (Fig. 5e; effect of fluid type: F(1,48) = 37.83, *P* < 0.001). Of most interest, there was an interaction between the bout range and the normalized volume consumed of each fluid type (Fig. 5f; F(7,48) = 7.645, *P* < 0.001), with greater normalized water consumption in small bouts, and greater normalized alcohol consumption in large bouts.

## Discussion

Accurate tracking of fluid consumption is important in preclinical studies of substance use, as well as psychopathology (e.g., sucrose preference test). The majority of prior studies have individually housed animals to measure a subject’s fluid intake, thereby modeling consumption in a potentially-stressful, non-naturalistic, socially isolated environment. Therefore, to study fluid consumption disambiguated from isolation, social tracking of fluid consumption is required, especially when wanting to understand social determinants of drinking behaviour. Here, we developed a fully open-source implementation of a robust solution to social fluid consumption tracking that was able to show individual differences in alcohol preference, circadian influence on drinking, and fluid type-dependent bout structure.

Firstly, while there have been prior iterations of open-source peristatic pumps [21, 22], our design presented here provides an alternative with remarkable accuracy. Using this device allowed us to track the volume consumed, rather than use a proxy for consumption such as licking. However, licking was still an important parameter, as we discovered a rat’s presence or beam-breaking was not adequate to define a drinking bout. Activation of the pump needed to correspond to licking to avoid false activation of the pump when a rat was near the RFID reader. We therefore used a capacitive lickometer on a chip design. Though infrared beam breaks have been used successfully as lickometers previously [23, 24], Petersen et al. have shown the superiority of capacitive systems over infrared as well as the improved flexibility of capacitive sensors on a chip compared to floor plate-based designs [25]. Thus, we were able to begin fluid delivery at a bout’s true start by activating the pump when a rat was both present and licking.

Pairing the lickometer with an Arduino-based microcontroller also allowed for time as an added dimension for fluid tracking. This enabled bout and circadian analysis of drinking behaviour. Rats were observed to do nearly all fluid consumption during the dark cycle as has been seen previously [26]. We were also able to show that rats tended to take larger bouts when drinking alcohol compared to water. Bout size may be an important measure related to excessive intake, with larger bout sizes in non-human primates being predictive of future heavy drinking [27], and greater bout sizes in high-drinking rat lines [28]. Quantity-frequency measures of alcohol consumption is also used in human diagnostics of regulatory loss of control [29].

The primary strength of using an RFID-based system is to track individual drinking bouts. FARESHARE was able to detect individual differences in drinking behaviour that would otherwise be lost if a simple group average was taken. One rat was observed to consume significantly more alcohol than its cage mates while another showed nearly no preference for alcohol over water, demonstrating FARESHARE’s ability to elucidate potential effects of rank, pretreatment, or individual substance vulnerability in a social context. As access is controllable via RFID, certain rats can have access restricted, allowing for control of partner rat consumption. Additionally, since the lickometer can still be active when access is restricted, extinction burst-like lick-spout activity measures may be taken when licking occurs without fluid delivery. Cage crowding can also be evaluated, as the system can handle as many animals as desired. FARESHARE is designed to be used in nearly any existing caging systems, therefore lacking the need to modify cages. As fluid reservoirs are outside the cage, frequent refilling can be avoided, preventing unwanted fluid loss, which is already reduced by active fluid delivery. Though the specific use cases might differ between labs requiring unique visual and statistical analyses, we have included R scripts in the supplementary material with our results as examples to help facilitate a variety of analyses with data collected through FARESHARE.

FARESHARE is not without limitations. Though we have tested FARESHARE with strawberry milkshake and up to 50% (w/w) sucrose with high accuracy, highly viscous fluids will not work properly due to the low diameter of the tubing used, leading to inaccurate volume measurements or pump failure. Based on our testing, this will only become a problem with very high-viscosity fluids such as honey. Additionally, at the time of this experiment, the software did not allow for the measurement of some common microstructure measurements such as interlick interval, interbout interval, bout duration, or the direct measure of lick frequency, however, this has now been updated and implemented. Though the pump durability is quite good for a non-commercial product, if FARESHARE is to be used for extended periods such as water tracking over several months, the tubing will likely require replacement every couple of months to ensure there is no failure. Another significant limitation is that while other animal species would benefit greatly from FARESHARE, it is currently limited to rats or other similar-sized rodents. Though FARESHARE was designed for rats to suit our specific use case, a mouse version could be implemented with minor modifications to the spout housing and programmed flow rate. With this open design, we hope to facilitate the creation of mouse-adapted devices in the near future. Another limitation is that the design’s current configuration precludes the use of neural monitoring/manipulation. Though the situation in which these techniques (optogenetics, calcium imaging, electrophysiology etc.) would be used in a group-housed setting is rare, this limitation can also be overcome in future iterations with altered RFID scanner and tag placement, or including the RFID in implant (e.g., our recently published open-source multi-electrode array design [30]). However, FARESHARE can already be used with chemogenetics, even for the purpose of providing delivery of chemogenetic ligands via the device, that can be controlled temporally or in an animal-specific manner.

We are excited to see how labs alter the design to fit their specific needs and overcome any limitations that they may encounter. We also hope that the low-cost will allow more labs to implement social tracking of fluid intake and study social determinants of substance use, as these important factors are often ignored in traditional methods and provide translational validity. In conclusion, accurate tracking of social fluid consumption in preclinical studies of substance use is crucial for understanding social determinants of drinking behaviour. Prior studies have often individually housed animals, potentially inducing stress and social isolation, which can confound the results and overlooks a major translationally relevant aspect of substance use development and treatment. To address this issue, a fully open-source implementation called FARESHARE was developed, enabling social fluid consumption tracking, and allowing researchers to explore individual differences in alcohol preference, circadian influence on drinking, and fluid type-dependent bout structure. FARESHARE has the potential to become an invaluable tool in unraveling the complex dynamics of substance use and psychopathology, offering new insights into the social determinants of drinking behaviour and facilitating the development of more effective interventions.

### Citation diversity statement

The authors have attested that they made efforts to be mindful of diversity in selecting the citations used in this article.

## Supplementary information

Supplementary Materials

## Data Availability

The data used for all figures and analyses are available at https://osf.io/ag74k/.The code, 3D printer files, and GERBER files for FARESHAREg are available at https://github.com/jfrie/FARESHARE.
