# Supplementary material for: FARESHARE: An open-source apparatus for assessing drinking microstructure in socially housed rats
Source: NPP Digit Psychiatry Neurosci. 2024 Feb 27;2:1. doi: 10.1038/s44277-024-00002-z (PMC12624943; doi:10.1038/s44277-024-00002-z)

Supplemental Material


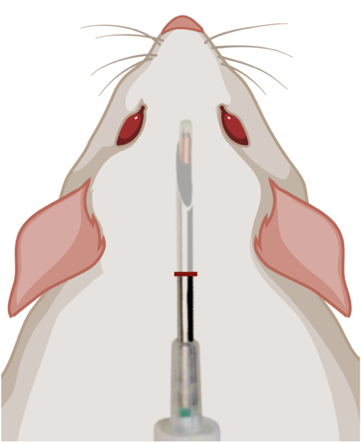


Supplementary Figure 1: Diagram of RFID positioning and injection sight.


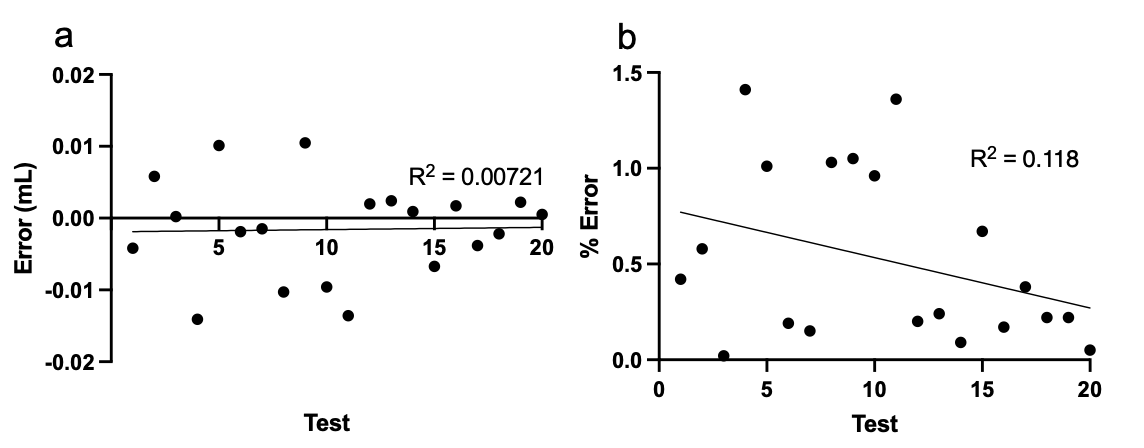


Supplementary Figure 2: Characterization of pump error in **a)** mL, and **b)** % error. Neither showed significant non-zero slope indicating consistent error across tests. Average pump error was 0.5% (95% CI 0.3-0.7).

Supplemental Figure 3: Representative raw output data for single rat. a) Cumulative volume consumed for Rat 1 across all 9 days. b) Total licks per bout for Rat 1 across all 9 days.


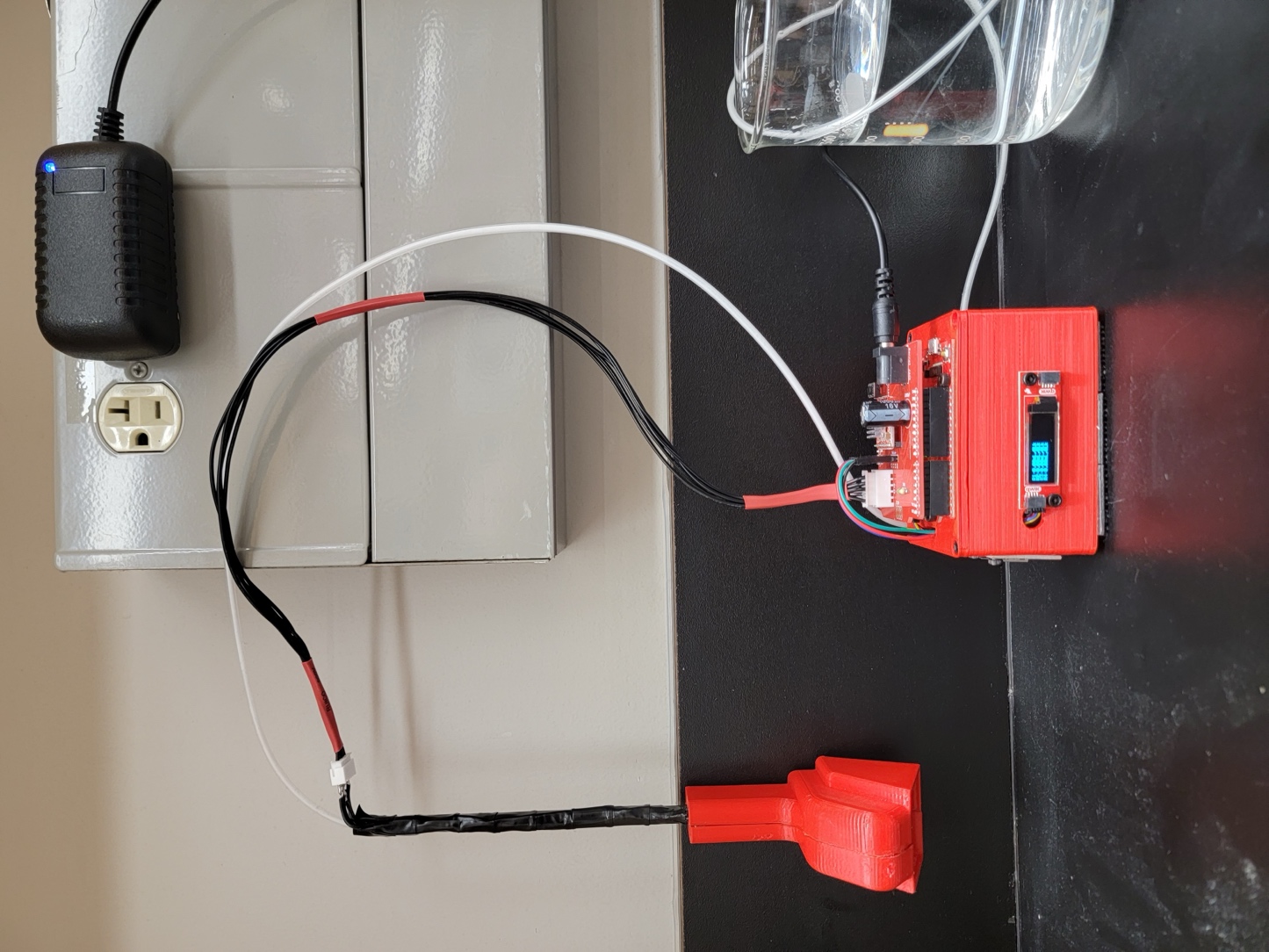


Supplemental Figure 4: Complete setup of device out side of cage.


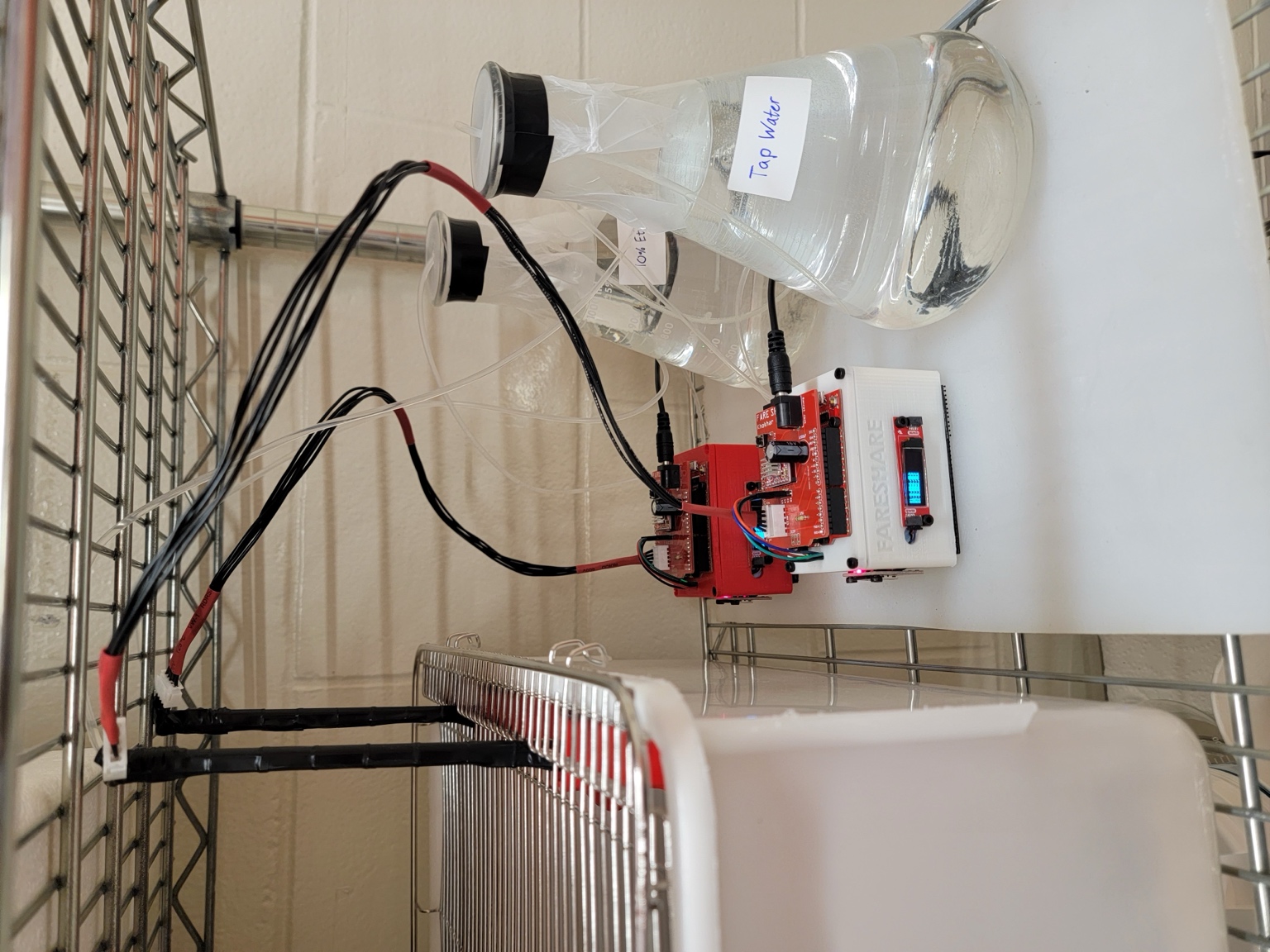


Supplemental Figure 5: Two devices set up in a group housed cage for a two-bottle-choice paradigm.

## Build Instructions

Table 1: Bill of Materials

| **Part Name** | **Distributer** | **Catalogue Number** | **Alternate** | **Cost ($US)** | |
| --- | --- | --- | --- | --- | --- |
| 1000uF Capacitor (through hole) | Mouser | 871-B43657A5108M057 | Digikey | | 1.63 |
| 16G Needles (x2) | Fisher Scientific | 1482618B | Surgo | | 0.85 |
| 1M Ohm Resistor (through hole) | Mouser | CFR-25JB-52-1M | Digikey | | 0.10 |
| 5-pin 2.54mm JST Female to Female Cable | Walmart | a14121800ux0572 | Amazon | | 1.30 |
| 5-pin 2.54mm JST Male header (x2) | Pololu | 2720 | Amazon | | 0.14 |
| 3D printer filament | Amazon | OVPLA175 | Hatchbox | | 2.50 |
| A4988 Stepper Motor Driver | Amazon | B07G15Z31B | Digikey | | 1.20 |
| Ball bearings (3mm x 8mm x 4mm) (x4) | Amazon | a19082100ux1557 | NewEgg | | 2.00 |
| Barrel Jack PCB mount (3 pin) | Mouser | 474-PRT-00119 | Digikey | | 1.25 |
| Break Away Headers (2.54mm straight) (41 pins) | Sparkfun | PRT-00116 | Mouser | | 3.50 |
| Custom PCB | JLCPCB | Custom Part | OSHpark | | 2.00 |
| Dual lock (10cm) | Amazon | B01G9EOEF2 | Mouser | | 1.50 |
| Epoxy | Walmart | 4200602 | Amazon | | 6.12 |
| Hook-up Wire (22 AWG) | Sparkfun | PRT-08022 | Amazon | | 2.95 |
| M3 Screw (Hex Socket, 25mm) (x4) | Amazon | a16040100ux1375 | Home Depot | | 2.75 |
| M3 Screw (Hex Socket, 10mm) (x7) | Amazon | a16040100ux1371 | Home Depot | | 3.25 |
| M3 Screw (Hex Socket, 5mm) (x8) | Amazon | a16042700ux1534 | Home Depot | | 3.50 |
| M3 Nut (x3) | Amazon | a16033100ux0563 | Home Depot | | 2.00 |
| Masterflex® Microbore Pump Tubing, Platinum-Cured Silicone, 1.42mm ID, 3.12mm OD (16cm long) | Avantor | MFLX95590-34 | Fisher Scientific | | 2.30 |
| Mini 4-pin Push Button | Amazon | B07VSNN9S2 | Walmart | | 1.00 |
| Micro SD Card (FAT16 or 32, 64MB to 32GB) | Amazon | B0912GL27G | Walmart | | 5.99 |
| Nema 17 Stepper Motor (4 lead, 2A) | Amazon | 17HS19-2004S1 | AliExpress | | 13.99 |
| Power Supply (12V, 3A) | Amazon | B0B51R6R2Y | Walmart | | 8.99 |
| Qwiic Cable Kit | Sparkfun | KIT-15081 | Mouser | | 8.95 |
| Qwiic OLED Display (0.91 in, 128x32) | Sparkfun | LCD-17153 | Mouser | | 10.95 |
| Qwiic OpenLog | Sparkfun | DEV-15164 | Mouser | | 18.50 |
| RedBoard Qwiic | Sparkfun | DEV-15123 | Mouser | | 21.50 |
| RFID Chip Injector (2.12x12mm) | AliExpress | ST-Y01 | Amazon | | 0.69 |
| RFID Glass Capsule (125kHz) | Sparkfun | SEN-09416 | Mouser | | 5.50 |
| RFID Reader Breakout | Sparkfun | SEN-13030 | Mouser | | 2.10 |
| RFID Reader ID-12LA (125 kHz) | Sparkfun | SEN-11827 | Mouser | | 32.50 |
| Silicone Tubing 1.5mm ID x 3mm (140 cm) | Amazon | a16090800ux0163 | NewEgg | | 6.99 |
| Stainless Steel Straw (straight 5mm OD) | Amazon | B099354XVF | Walmart | | 1.00 |
| **Total** |  |  |  | | **$179.49** |

PCB Ordering: Upload GERBER file to <https://jlcpcb.com> and order with default settings.

PCB Soldering: The figure below shows the PCB before (left) and after (right) soldering.


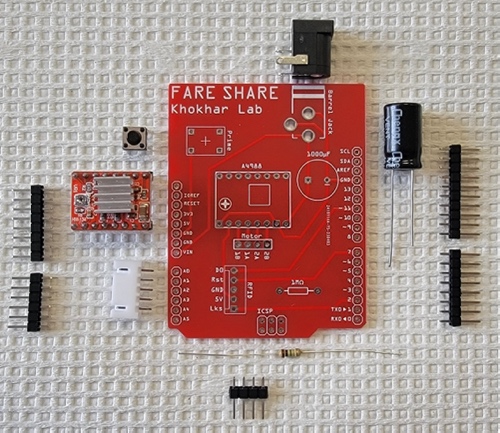

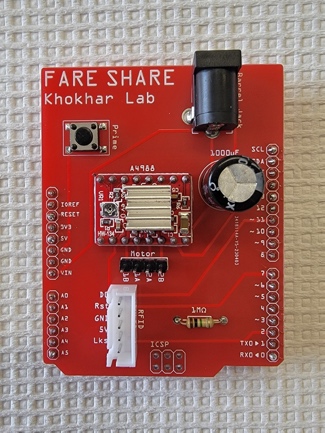


3D Printing: Print in the orientations shown below. The pump rotor and RFID housing should be printed with supports. All other components can be printed without supports. Print with standard PLA filament.


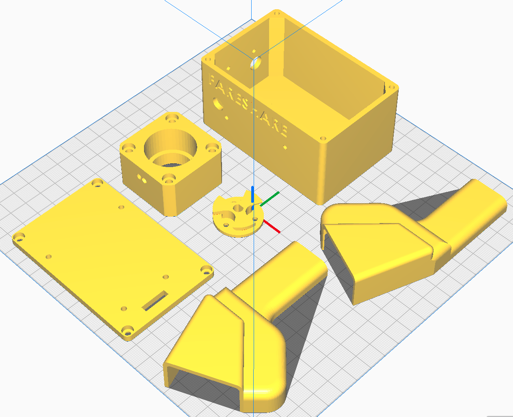


Pump Assembly:

1. Cut two 1cm pieces of needle slowly rotating and cutting evenly so as not to crimp.


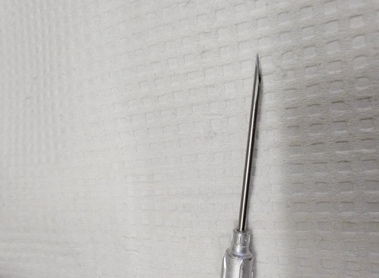

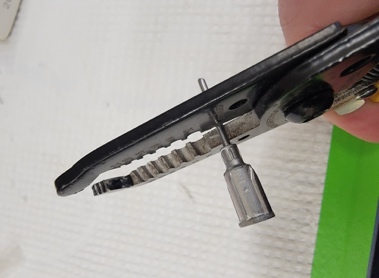

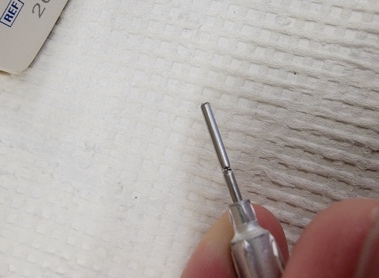

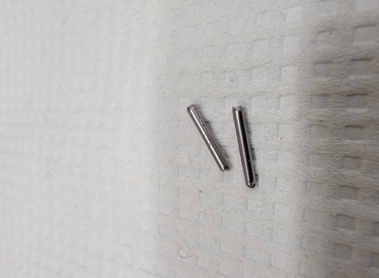


1. Screw M3X10 screws through the holes of the rotor


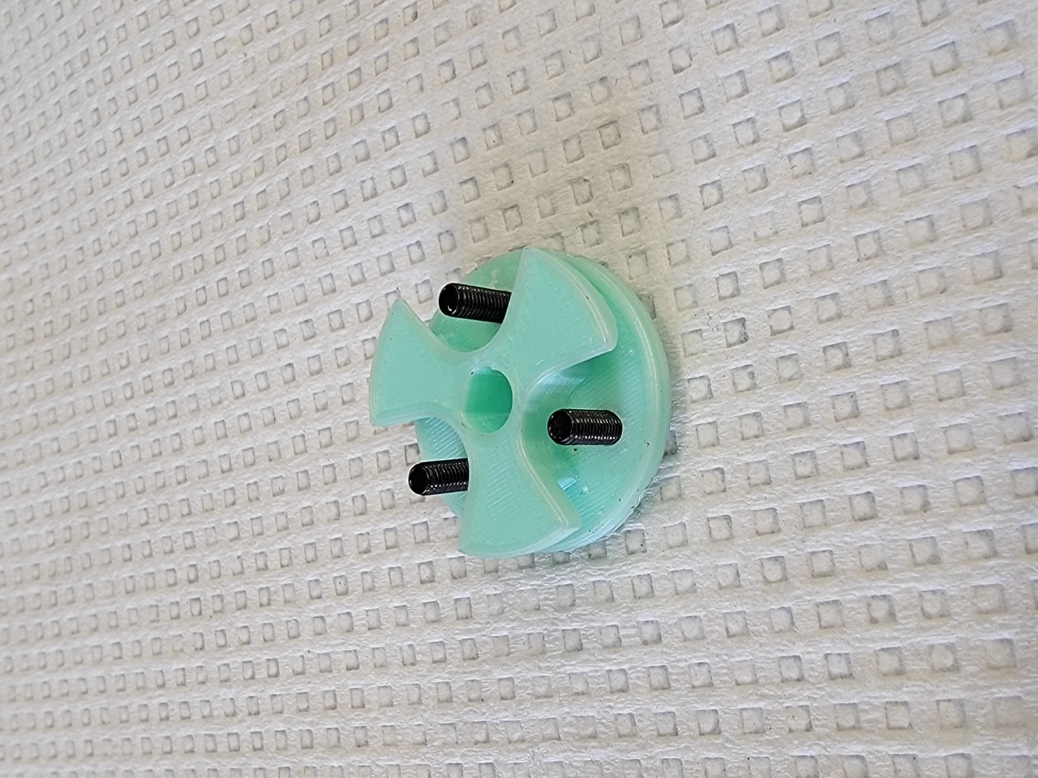


1. Place bearings on screws and secure with M3 nuts


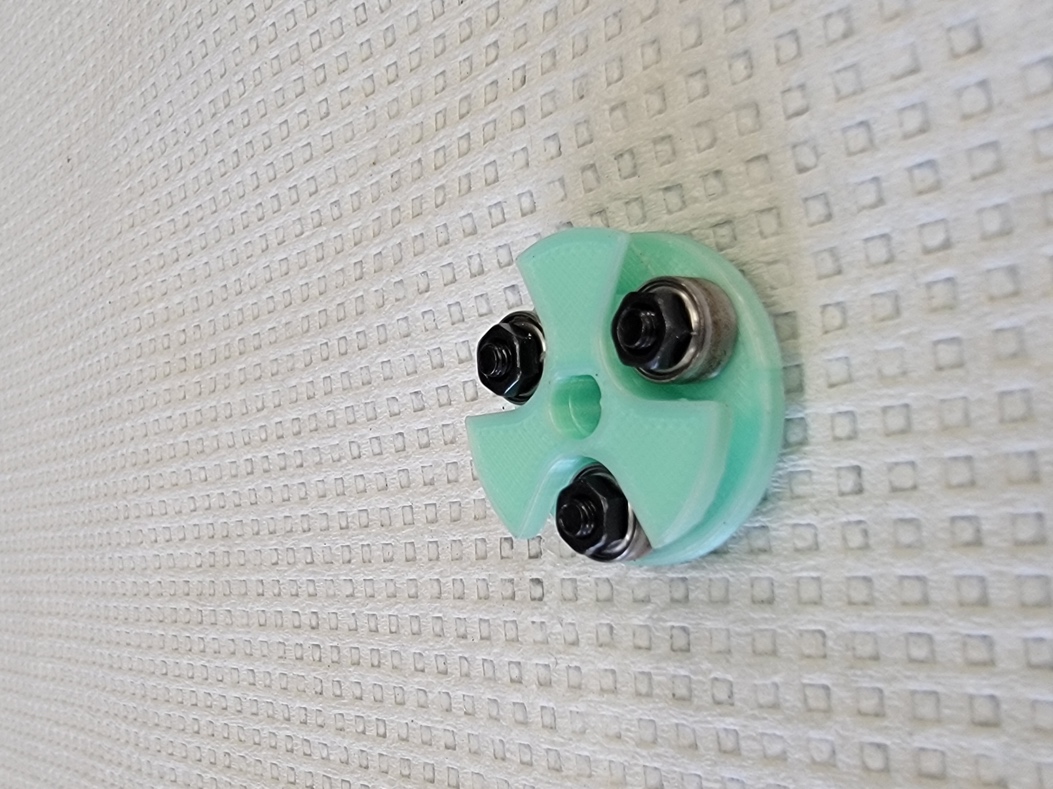


1. Thread 15cm segment of Masterflex tubing through pump case.
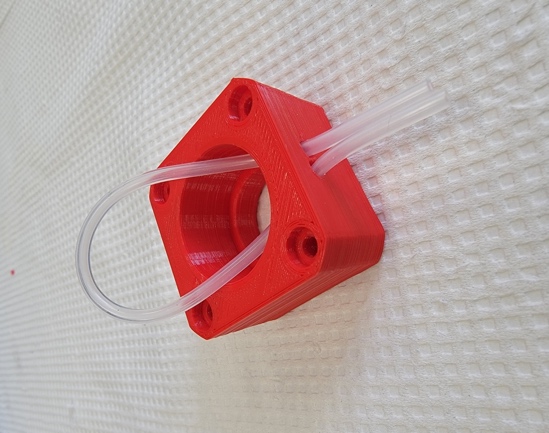

2. Wrap the tubing around the rotor and push the rotor into the pump housing while keeping tension on the tubing. Once inside the housing, manually rotate the rotor a bit and pull on each side of the tubing until the rotor is straight and the tubing wraps around each bearing.


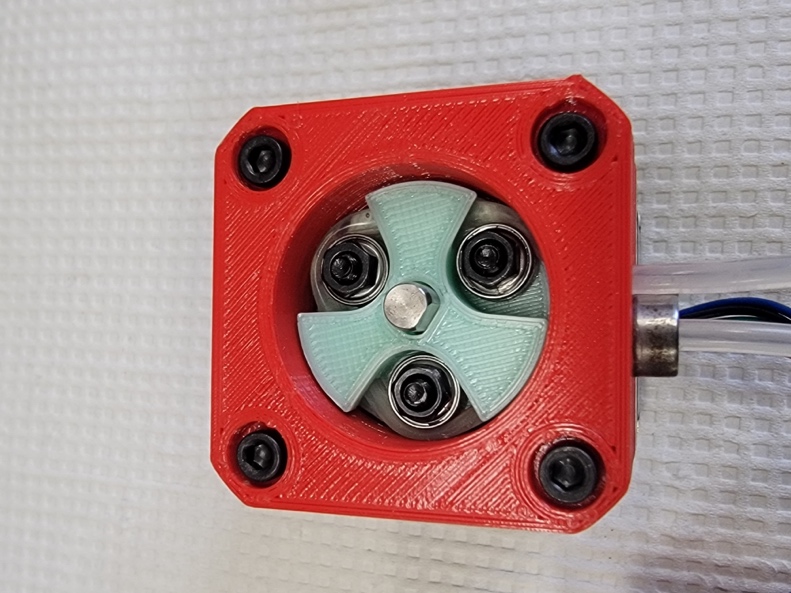


1. Thread another bearing onto the left (with tubing facing you) exiting tube.


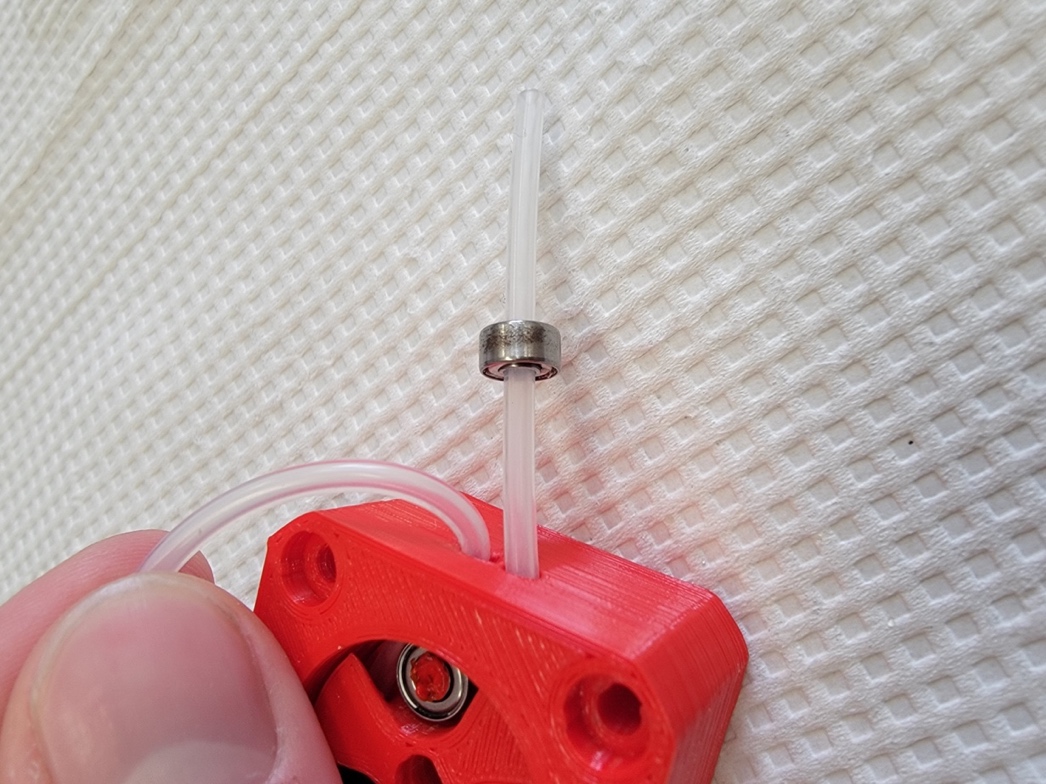


1. Bring bearing up to pump housing and epoxy to both tubing and housing. This will keep the tubing from being pulled through the rotor or shifting.


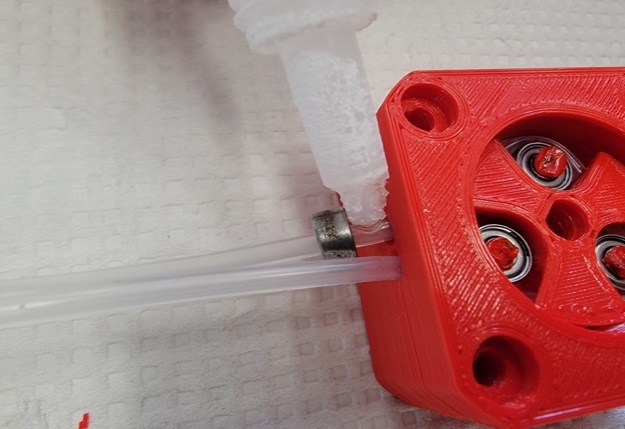

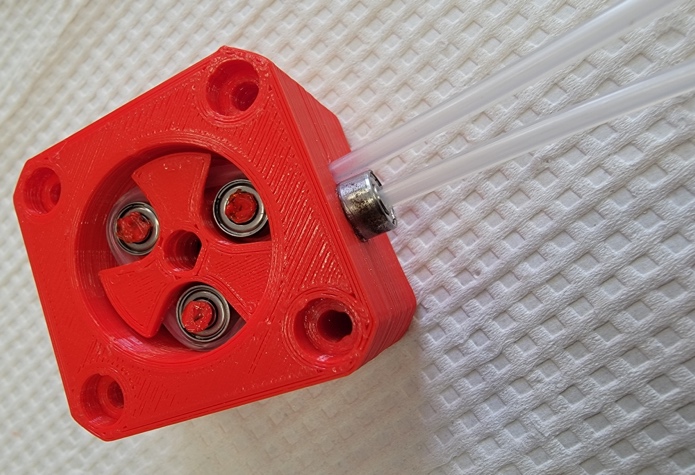


1. Insert needle segments halfway into tubing ends.


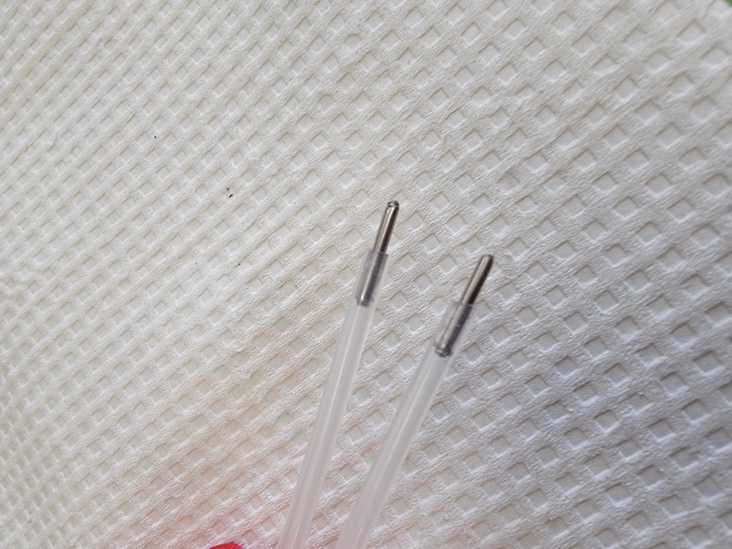


1. Place super glue where needle exits tubing and insert needle ends into two 70 cm segments of silicon tubing (cheaper tubing), gluing tubes together.


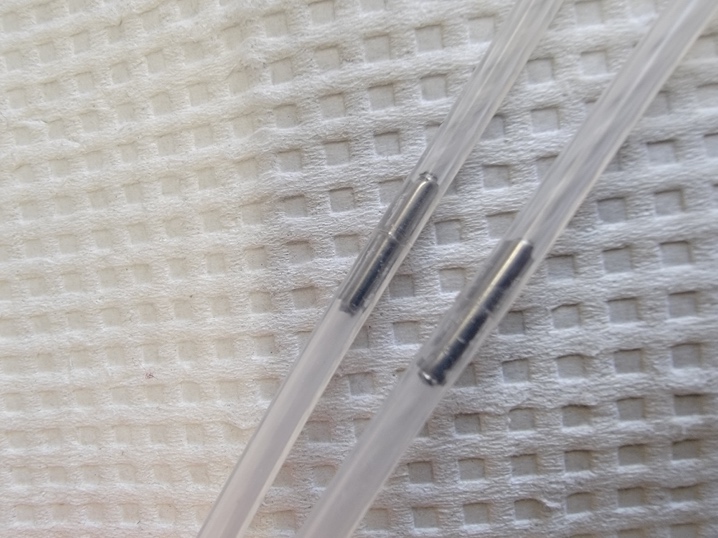


1. Place pump assembly onto motor with tubing facing the same direction as motor wires and, using M3x25mm screws, fix in place.


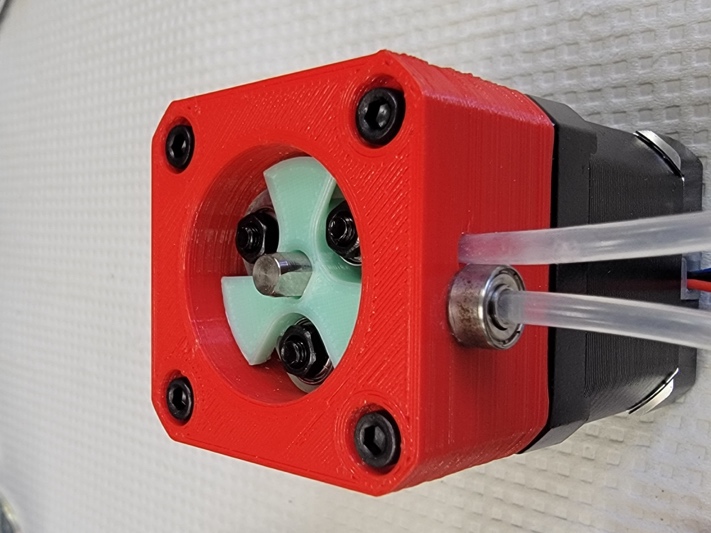


1. Place assembly into box, feeding tubing through input and output holes, and wrapping most of the motor wiring together, placing it in the space between the motor and the wall of the box. Spray the bearings and inner tubing generously with an all-purpose lubricant.


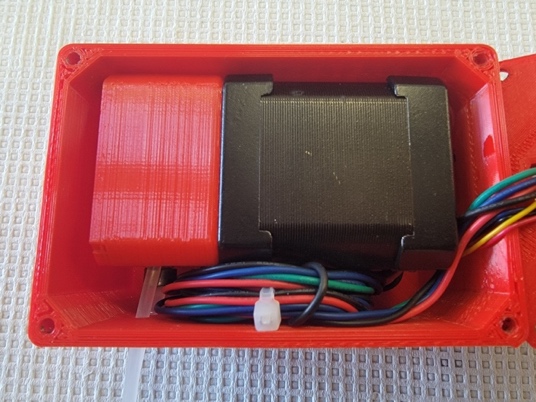


1. Attach OLED display to side of box with M3x4mm screws and thread 10cm Qwiic cable from the left port on the OLED into box.


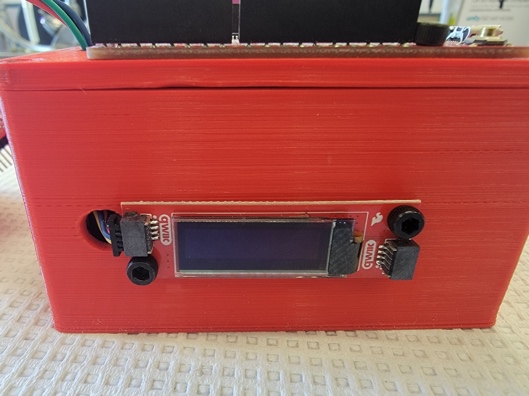


1. Attach data logger to the side of the box with M3x4mm screws and connect Qwiic cable from OLED into right port of data logger. Connect left port to 5cm Qwiic cable.


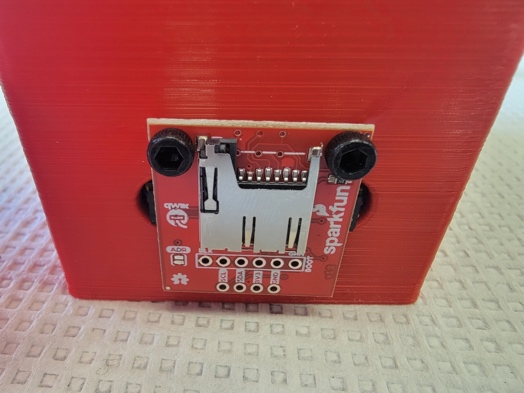


1. Attach RedBoard to box lid with M3x4mm screws. Feed motor wiring and Qwiic cable from data logger through square channel in lid. Connect Qwiic cable to RedBoard. Connect lid to box with M3x10mm screws.


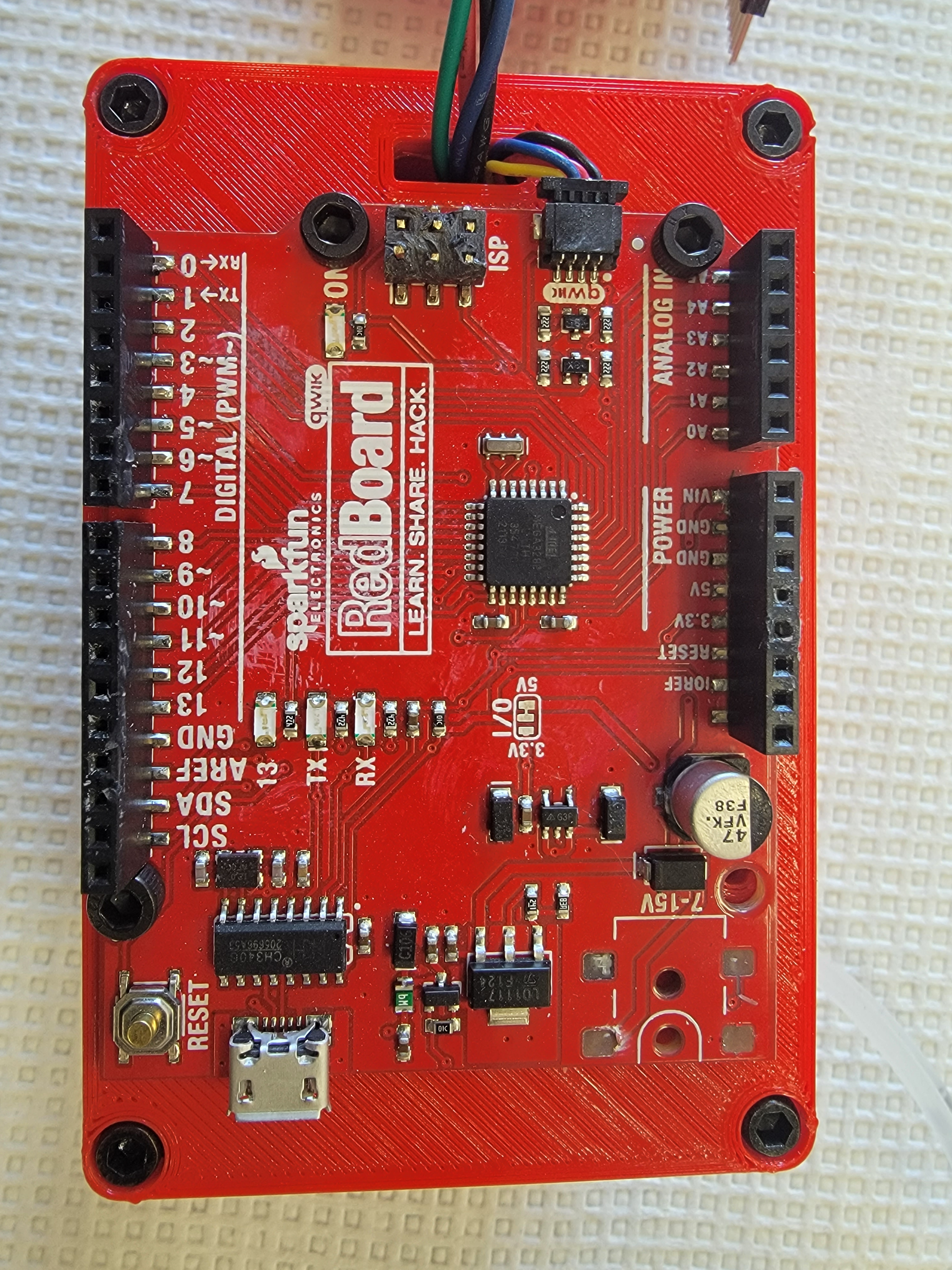


1. Plug PCB into RedBoard and connect motor wires to PCB motor pins with the black wire closest to the resistor.


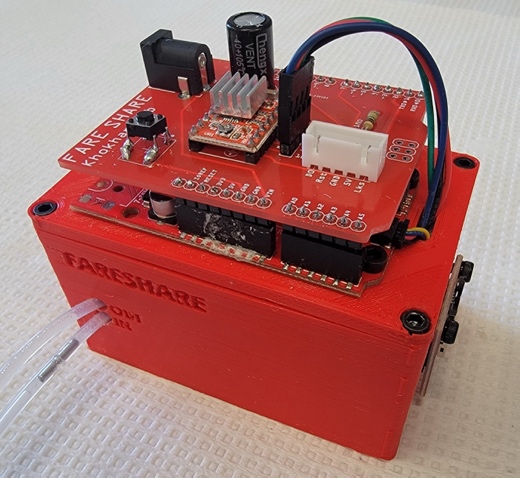


RFID Housing Assembly:

1. Solder RFID Breakout Board to RFID Reader.


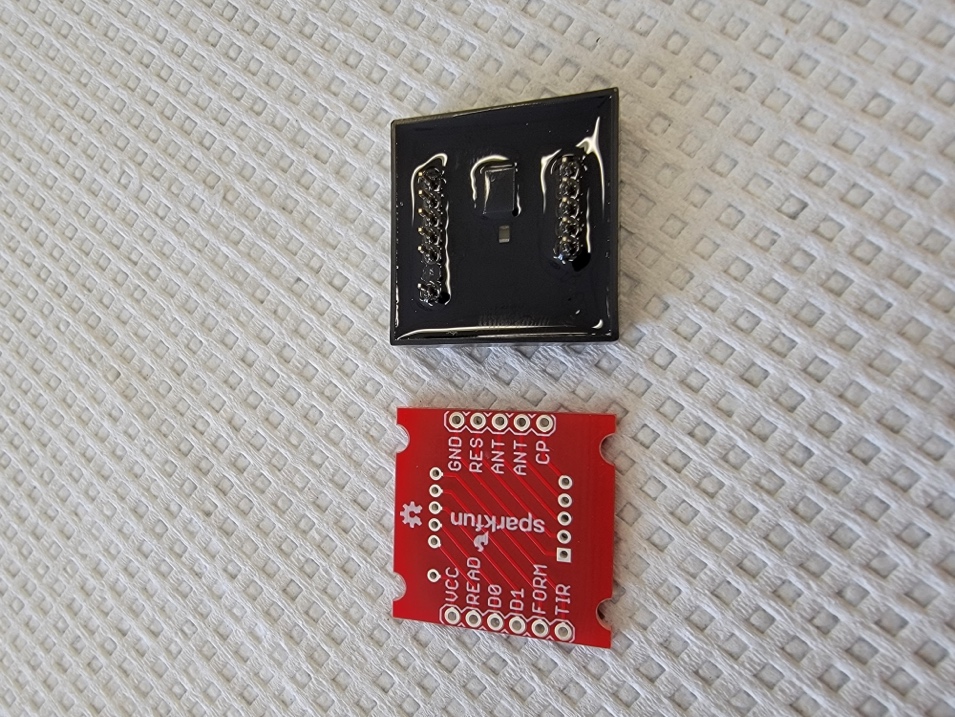

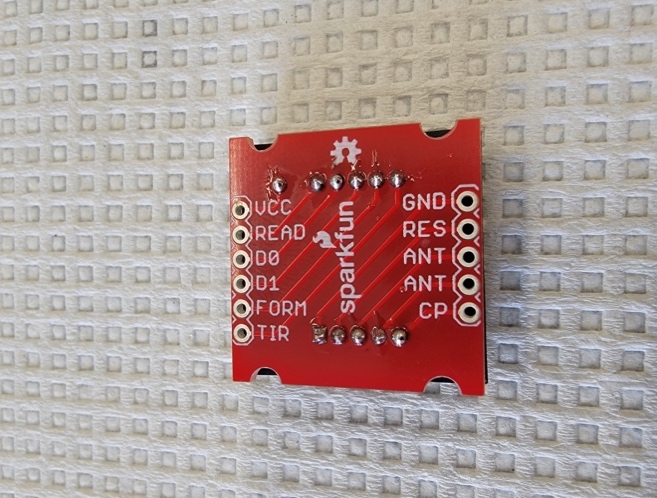


1. Solder a wire between the FORM and GND pins.


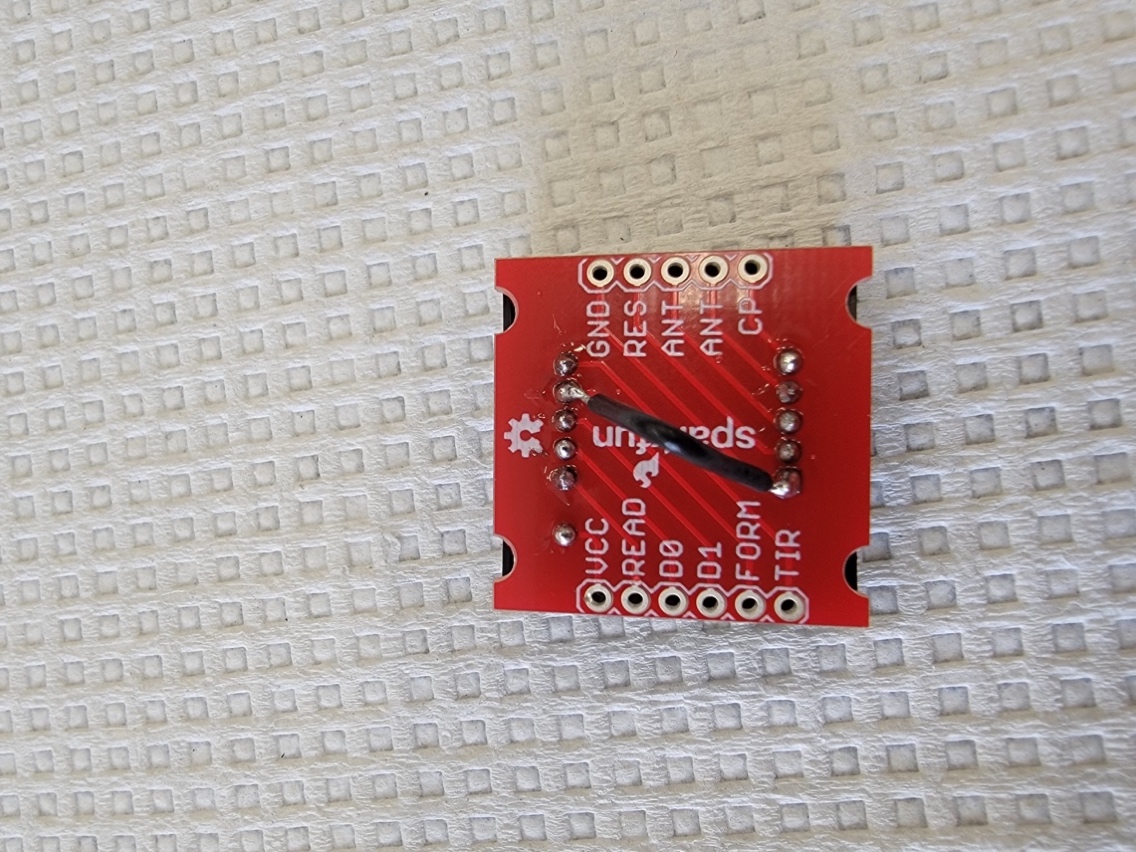


1. Solder 30cm segments of wire to Vcc, D0, GND, and RST through hole pins. Label these wires at the unattached ends so you can identify them later on once they are encased.


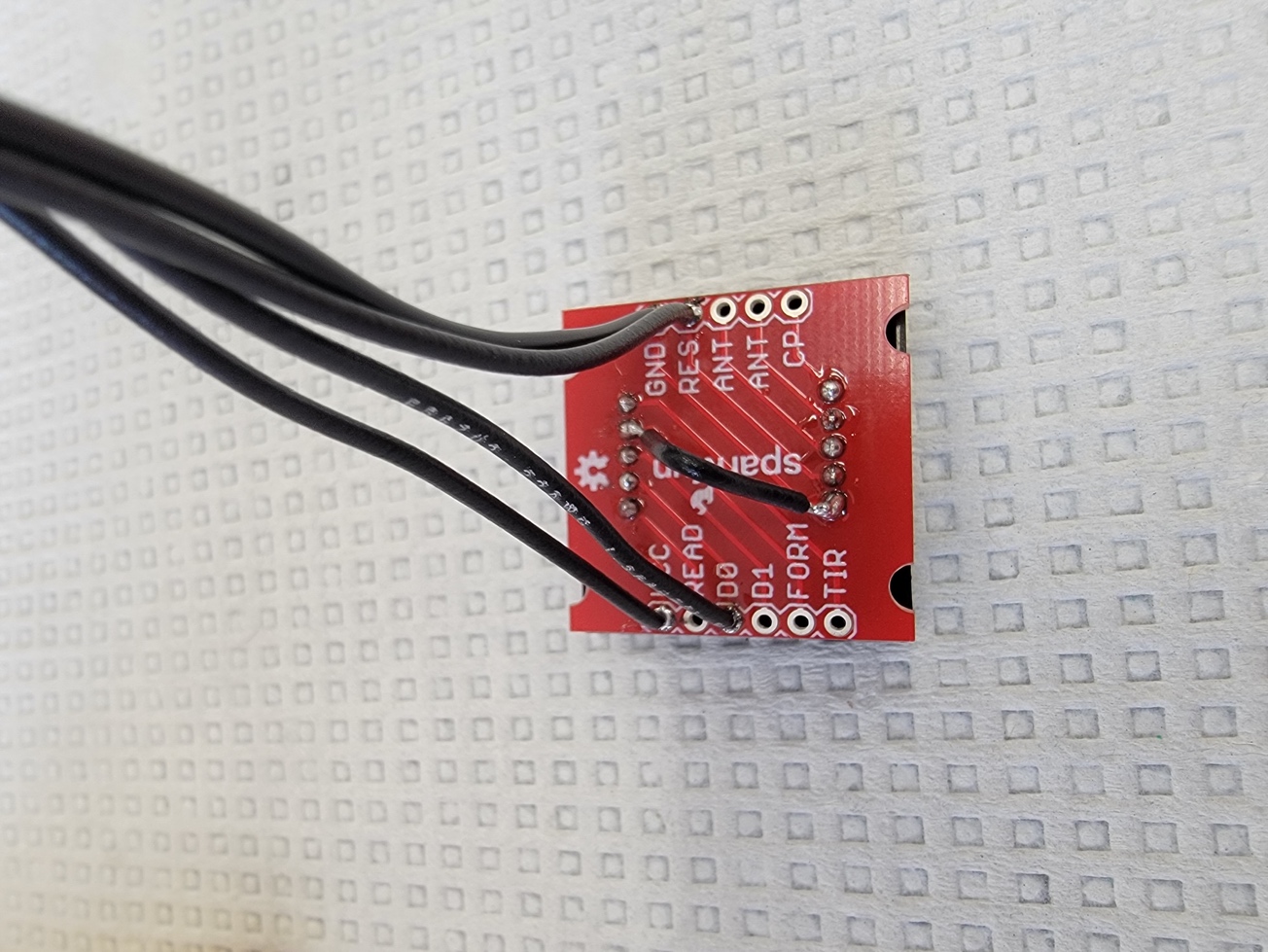


1. Epoxy the metal straw into one half of the RFID housing with 0.5cm of straw projecting past the straw channel. Using glue or command strips, secure RFID sensor inside housing and thread wires through wire channel. Epoxy the other half of RFID housing together.


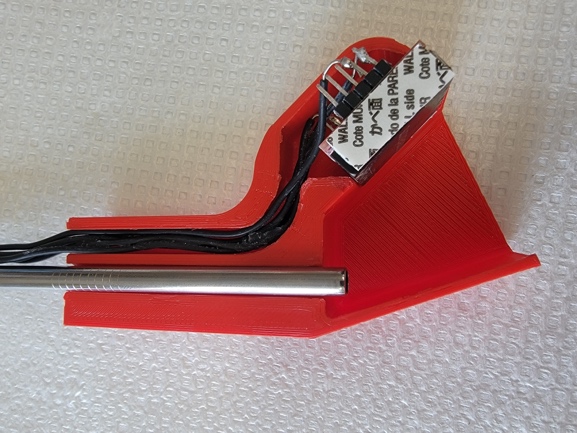


1. Cut the wires such that they are 2cm longer than the straw and, with the same orientation as the plugin on the PCB, solder each wire to a JST header.


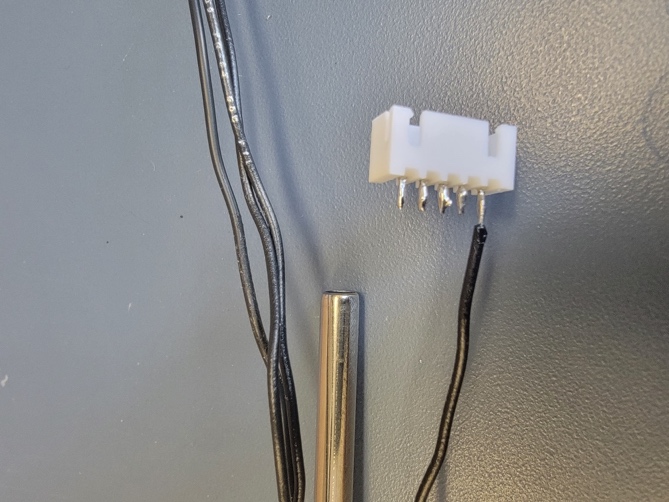

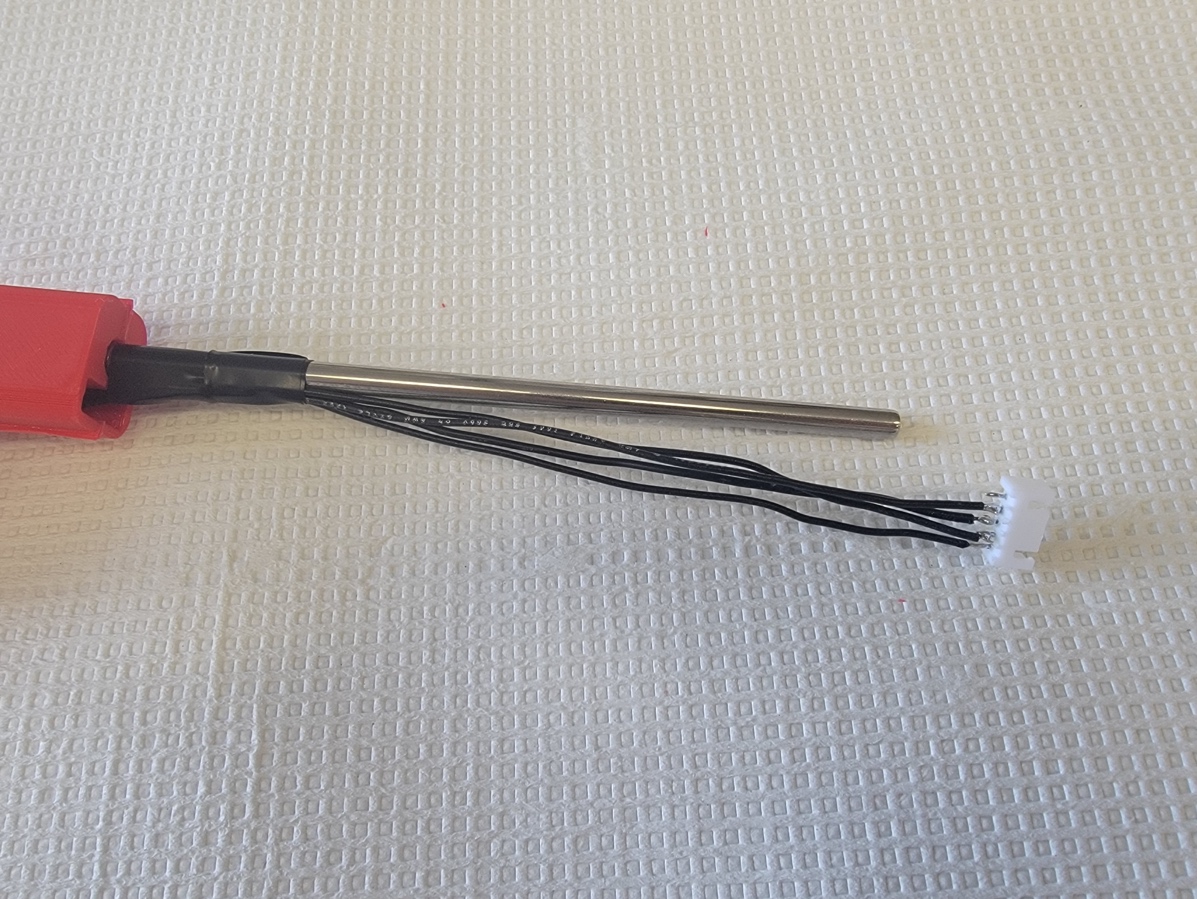


1. Strip 15cm off a 20cm wire. Wrap the stripped section around the tip of the straw and secure with electrical tape. Cut the wire to line up with the remaining pin on the JST header and solder.


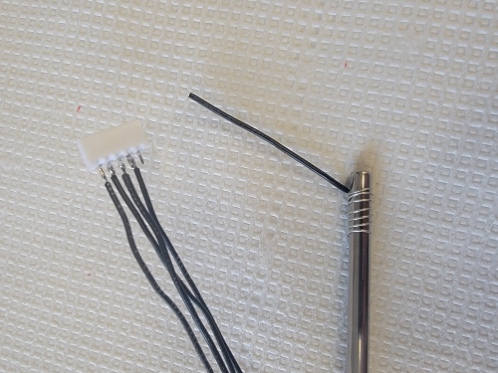

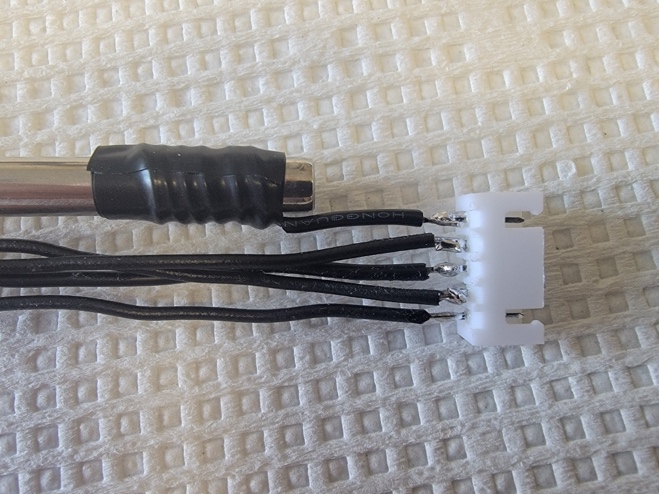


1. Use electrical tape to secure all wire to the straw.


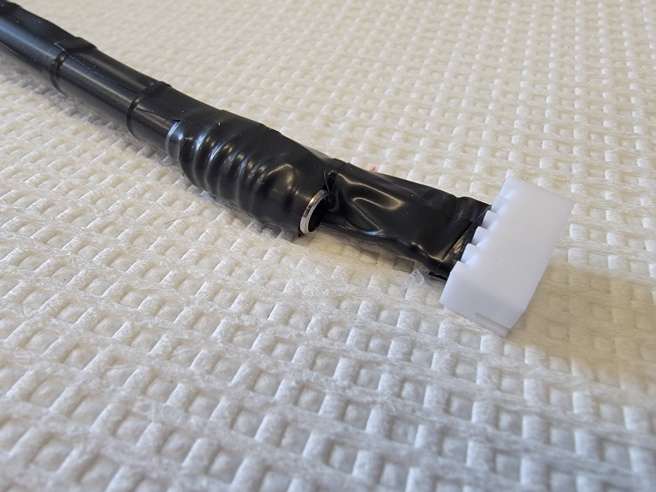

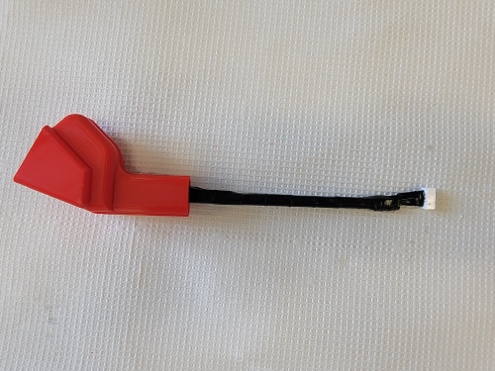


Load libraries: In the Arduino IDE select Tools>Manage Libraries. In the library manager, download Adafruit GFX Library, Adafruit SSD1306, SparkFun Qwiic OpenLog, and CapacitiveSensor.

Addition of RFID Tags to Code: Upload FARESHARE.ino to RedBoard Qwiic. Open serial monitor and place new RFID tag under RFID sensor. The serial monitor will display the new tag ID. Copy this ID and use it to replace the ID on line 38 of the code. Do this for each tag in the following lines for as many rats as are being tracked. Add one additional tag that will be used for priming the pump tubing with fluid when starting an experiment. **Note:** FARESHARE can track as many animals as needed; however, the OLED display only has room to show the overall results of the first four rats.

Pump Calibration: Each peristaltic pump will be slightly different. Therefore, to ensure each pump delivers an accurate volume and at the same rate, the pumps must be calibrated by the following instructions:

1. Place the input tube into a container of water and the output into a beaker that is at least 10mL.
2. Prime the line using the push button.
3. Place the beaker with the output tube onto an analytical scale.
4. Place an RFID tag by the RFID sensor and tap the straw with your finger to activate the motor. Time how long it takes for FARESHARE to deliver 10g of water.
5. Plug this time in seconds into the following formula: $Scale_{motor}=\frac{420}{time}$ and set the motor_scale variable on line 83 to this new value and upload the new code. This will ensure each device takes the same amount of time to dispense fluid (~7 minutes for 10mL).
6. Tare the scale and fill it to 10g again with the pump.
7. Plug the volume reading on the FARESHARE OLED display into the following formula: $Scale_{flow}=\frac{10}{volume}$ and set the flow_scale variable on line 81 to this new value and upload the new code.

## Operating instructions

1. Prior to starting experiments, remove any .csv or .txt files from the SD card. Insert the SD card into the SD card slot on the Qwiic OpenLog. **Note:** The Qwiic OpenLog SD card logger used in FARESHARE is compatible with 64MB to 32GB microSD cards in either FAT16 or FAT32 formats.
2. Place a strip of 3M type 400 dual lock on the back of the 3D printed RFID housing. Place another strip on the inside of the cage where you want the device to sit. Lock device in place and feed straw through cage top wire.
3. Connect wires to PCB via 5-pin JST cable.
4. Place input tube into fluid reservoir and tape in place
5. Plug power supply into the wall and connect the barrel connector to the barrel jack on the PCB. **Note:** Ensure SD card is in reader prior to plugging as files are created when device powers up.
6. Press the reset button on the Arduino to begin the experiment. **Note:** make a note of the time that the experiment is started as the timestamps for each drinking bout are measured in milliseconds following this moment.
7. Place the priming tag onto the RFID sensor to operate the pump until fluid has fully filled the line.
8. Place output tube ~6cm into straw and tape in place.
9. Allow rats to drink as long as desired.
10. When experiment is complete, unplug FARESHARE and remove SD card.

FARESHARE Analysis Scripts

## Script 1

Loads libraries and combines all .csv output files from FARESHARE into one dataframe. Run this before all other scripts.

#Load libraries
library(dplyr);
library(ggplot2);
library(lubridate);
library(tidyr);
library(emmeans);
library(multcomp);

# Read the CSV files into data frames
rat1alc <- read.csv("rat1_alcohol.csv")
rat2alc <- read.csv("rat2_alcohol.csv")
rat3alc <- read.csv("rat3_alcohol.csv")
rat4alc <- read.csv("rat4_alcohol.csv")
rat1wat <- read.csv("rat1_water.csv")
rat2wat <- read.csv("rat2_water.csv")
rat3wat <- read.csv("rat3_water.csv")
rat4wat <- read.csv("rat4_water.csv")

# Add a two new columns to each data frame to indicate the fluid type and rat number
rat1alc$fluid <- basename("alcohol")
rat1alc$rat_num <- basename("1")
rat2alc$fluid <- basename("alcohol")
rat2alc$rat_num <- basename("2")
rat3alc$fluid <- basename("alcohol")
rat3alc$rat_num <- basename("3")
rat4alc$fluid <- basename("alcohol")
rat4alc$rat_num <- basename("4")
rat1wat$fluid <- basename("water")
rat1wat$rat_num <- basename("1")
rat2wat$fluid <- basename("water")
rat2wat$rat_num <- basename("2")
rat3wat$fluid <- basename("water")
rat3wat$rat_num <- basename("3")
rat4wat$fluid <- basename("water")
rat4wat$rat_num <- basename("4")

# Combine the data frames into one long data frame
df <- rbind(rat1alc, rat2alc, rat3alc, rat4alc, rat1wat, rat2wat, rat3wat, rat4wat)

## Script 2

Creates a graph of the cumulative volume of each substance consumed by a single subject.

# Filter data for subject 1
df_subject1 <- df %>%
 filter(rat_num == 1)

# Convert Start.Time..ms. to hours
df_subject1 <- df_subject1 %>%
 mutate(start_time_hr = Start.Time..ms. / 3600000)

# Calculate cumulative volume for each fluid type
df_subject1_cumulative <- df_subject1 %>%
 arrange(fluid, start_time_hr) %>%
 group_by(fluid) %>%
 mutate(cumulative_volume = cumsum(Bout.Volume..mL.))

# Plot cumulative volume over time
ggplot(df_subject1_cumulative, aes(x = start_time_hr, y = cumulative_volume, color = fluid)) +
 geom_line() +
 scale_color_manual(values = c("water" = "blue", "alcohol" = "orange")) +
 labs(x = "Time (hours)", y = "Cumulative Volume (mL)", color = "Fluid Type") +
 theme_minimal() + theme(legend.title=element_blank())


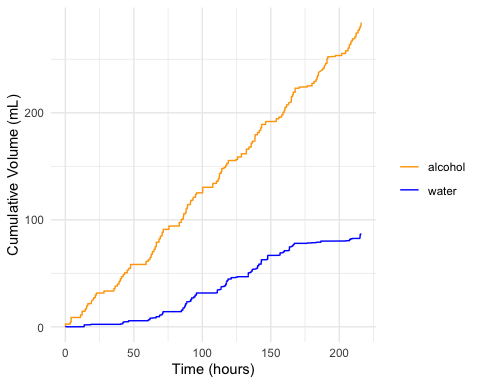


## Script 3

Creates a plot of licks in a bout over time for a single subject.

# Convert Start.Time..ms.s to hours
df$start_time_hr <- df$Start.Time..ms. / (1000 * 60 * 60)

# Filter data for subject 1
df_subject1 <- df[df$rat_num == 1, ]

# Create the plot
ggplot(df_subject1, aes(x = start_time_hr, y = Bout.Licks, color = fluid)) +
 geom_segment(aes(xend = start_time_hr, yend = 0), linewidth = 1) +
 scale_color_manual(values = c("water" = "blue", "alcohol" = "orange")) +
 theme_minimal() +
 labs(x = "Time (hours)", y = "Lick Number", color = "Fluid Type") +
 theme(legend.position = "right") + theme(legend.title=element_blank())


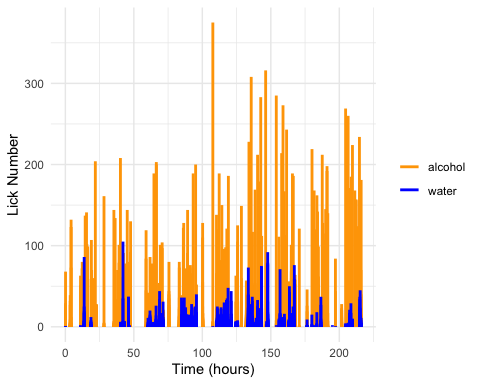


## Script 4

Graphs average drinking in a given hour of the day over a 24 hour period for a single subject. Runs an ANOVA on data and compare each hour. Plots stars on significant comparisons.

df$Start.Time..ms. <- as.numeric(df$Start.Time..ms.)
df$End.Time..ms. <- as.numeric(df$End.Time..ms.)
df$Bout.Volume..mL. <- as.numeric(df$Bout.Volume..mL.)
df$Bout.Licks <- as.numeric(df$Bout.Licks)
df$fluid <- as.factor(df$fluid)
df$rat_num <- as.factor(df$rat_num)

# Remove unused columns
df_needed <- df[,-c(2, 4)]

# Choose which subject here (currently rat 1)
df_filtered <- subset(df_needed, rat_num == 1)

# Get hour and day from time data
df_filtered$Hour <- (as.numeric(df_filtered$Start.Time..ms.) %% (24 * 60 * 60 * 1000)) / (60 * 60 * 1000)
df_filtered$Hour <- floor(df_filtered$Hour)
df_filtered$Hour <- df_filtered$Hour + 1
df_filtered$Day <- as.numeric(df_filtered$Start.Time..ms.) %/% (24 * 60 * 60 * 1000) + 1

# Remove unused column
df_filtered <- df_filtered[,-4]

# Sum volume by day, hour, and fluid, and deal with hours with no data
df_summed <- df_filtered %>%
 group_by(Day, Hour, fluid) %>%
 summarise(Total.Volume = sum(Bout.Volume..mL., na.rm = TRUE), .groups = 'drop') %>%
 complete(Day, Hour, fluid, fill = list(Total.Volume = 0))

# Calculate average volume by hour and fluid across all days
df_average <- df_summed %>%
 group_by(Hour, fluid) %>%
 summarise(Avg.Volume = mean(Total.Volume), .groups = 'drop')

# Calculate SEM (Standard Error of the Mean)
df_average_sem <- df_summed %>%
 group_by(Hour, fluid) %>%
 summarise(Avg.Volume = mean(Total.Volume),
 SEM = sd(Total.Volume) / sqrt(n()), .groups = 'drop')

# Convert factors for ANOVA
df_summed$Hour <- as.factor(df_summed$Hour)
df_summed$fluid <- as.factor(df_summed$fluid)

# ANOVA with interaction between fluid and hour
anova_result <- aov(Total.Volume ~ fluid * Hour, data = df_summed)
summary(anova_result)

## Df Sum Sq Mean Sq F value Pr(>F)
## fluid 1 98.4 98.38 86.678 < 2e-16 ***
## Hour 21 166.7 7.94 6.994 < 2e-16 ***
## fluid:Hour 21 62.1 2.96 2.606 0.000178 ***
## Residuals 352 399.5 1.13
## ---
## Signif. codes: 0 '***' 0.001 '**' 0.01 '*' 0.05 '.' 0.1 ' ' 1

# Pairwise comparisons of fluid at each hour
emmeans_result <- emmeans(anova_result, pairwise ~ fluid | Hour)
pairs(emmeans_result)

## Hour = 1:
## contrast estimate SE df t.ratio p.value
## alcohol - water 0.2836 0.502 352 0.565 0.5727
##
## Hour = 2:
## contrast estimate SE df t.ratio p.value
## alcohol - water -0.1463 0.502 352 -0.291 0.7710
##
## Hour = 3:
## contrast estimate SE df t.ratio p.value
## alcohol - water 0.4307 0.502 352 0.858 0.3917
##
## Hour = 4:
## contrast estimate SE df t.ratio p.value
## alcohol - water 0.0901 0.502 352 0.179 0.8577
##
## Hour = 5:
## contrast estimate SE df t.ratio p.value
## alcohol - water 1.3309 0.502 352 2.650 0.0084
##
## Hour = 6:
## contrast estimate SE df t.ratio p.value
## alcohol - water 0.3612 0.502 352 0.719 0.4724
##
## Hour = 9:
## contrast estimate SE df t.ratio p.value
## alcohol - water 0.4001 0.502 352 0.797 0.4262
##
## Hour = 10:
## contrast estimate SE df t.ratio p.value
## alcohol - water 0.5009 0.502 352 0.997 0.3192
##
## Hour = 11:
## contrast estimate SE df t.ratio p.value
## alcohol - water 0.3191 0.502 352 0.635 0.5256
##
## Hour = 12:
## contrast estimate SE df t.ratio p.value
## alcohol - water 1.3879 0.502 352 2.764 0.0060
##
## Hour = 13:
## contrast estimate SE df t.ratio p.value
## alcohol - water 1.6434 0.502 352 3.272 0.0012
##
## Hour = 14:
## contrast estimate SE df t.ratio p.value
## alcohol - water -0.2909 0.502 352 -0.579 0.5628
##
## Hour = 15:
## contrast estimate SE df t.ratio p.value
## alcohol - water 1.9626 0.502 352 3.908 0.0001
##
## Hour = 16:
## contrast estimate SE df t.ratio p.value
## alcohol - water 1.5749 0.502 352 3.136 0.0019
##
## Hour = 17:
## contrast estimate SE df t.ratio p.value
## alcohol - water 2.9313 0.502 352 5.837 <.0001
##
## Hour = 18:
## contrast estimate SE df t.ratio p.value
## alcohol - water 0.8945 0.502 352 1.781 0.0758
##
## Hour = 19:
## contrast estimate SE df t.ratio p.value
## alcohol - water 1.4292 0.502 352 2.846 0.0047
##
## Hour = 20:
## contrast estimate SE df t.ratio p.value
## alcohol - water 0.9144 0.502 352 1.821 0.0695
##
## Hour = 21:
## contrast estimate SE df t.ratio p.value
## alcohol - water 1.3425 0.502 352 2.673 0.0079
##
## Hour = 22:
## contrast estimate SE df t.ratio p.value
## alcohol - water 1.5625 0.502 352 3.111 0.0020
##
## Hour = 23:
## contrast estimate SE df t.ratio p.value
## alcohol - water 0.8441 0.502 352 1.681 0.0937
##
## Hour = 24:
## contrast estimate SE df t.ratio p.value
## alcohol - water 2.1641 0.502 352 4.309 <.0001

# Get the summary of pairwise comparison results
pairwise_summary <- summary(pairs(emmeans_result))

# Extract significant hours
significant_hours <- pairwise_summary[pairwise_summary$p.value < 0.05, ]
unique_significant_hours <- unique(significant_hours$Hour)

# Get max average volume for y-axis limit
max_y <- max(df_average_sem$Avg.Volume + df_average_sem$SEM, na.rm = TRUE)

# Line plot with SEM error bands, black stars for significant hours, updated axis titles, background changes, no title, and x-axis limits
p <- ggplot(df_average_sem, aes(x = Hour, y = Avg.Volume, color = fluid)) +
 geom_rect(aes(xmin = 12, xmax = 24, ymin = -Inf, ymax = Inf), fill = "grey90", color = NA) +
 geom_line() +
 geom_ribbon(aes(ymin = Avg.Volume - SEM, ymax = Avg.Volume + SEM, fill = fluid), alpha = 0.2) +
 scale_color_manual(values = c("alcohol" = "orange", "water" = "blue")) +
 scale_fill_manual(values = c("alcohol" = "orange", "water" = "blue")) +
 scale_x_continuous(breaks = 1:24, limits = c(1, 24))

# Add black stars to the plot
for (range in unique_significant_hours) {
 test <- df_average_sem %>%
 filter(Hour == range)
 if (length(unique_significant_hours) > 0) {
 p <- p + geom_text(data = test, aes(label = "*", y = max(Avg.Volume+SEM) *1.075), size = 8, vjust = 0, color = "black")

 }
}

# Final plot adjustments with updated axis titles, background changes, no title, and x-axis limits
p + theme_minimal() +
 theme(panel.grid = element_blank(), panel.border = element_blank(), panel.background = element_blank(), plot.title = element_blank()) +
 labs(x = "Hour (ZT)", y = "Average Hourly Volume (mL)", color = "Fluid Type", fill = "Fluid Type") +
 ylim(0, max_y * 1.1) + theme(legend.title=element_blank())


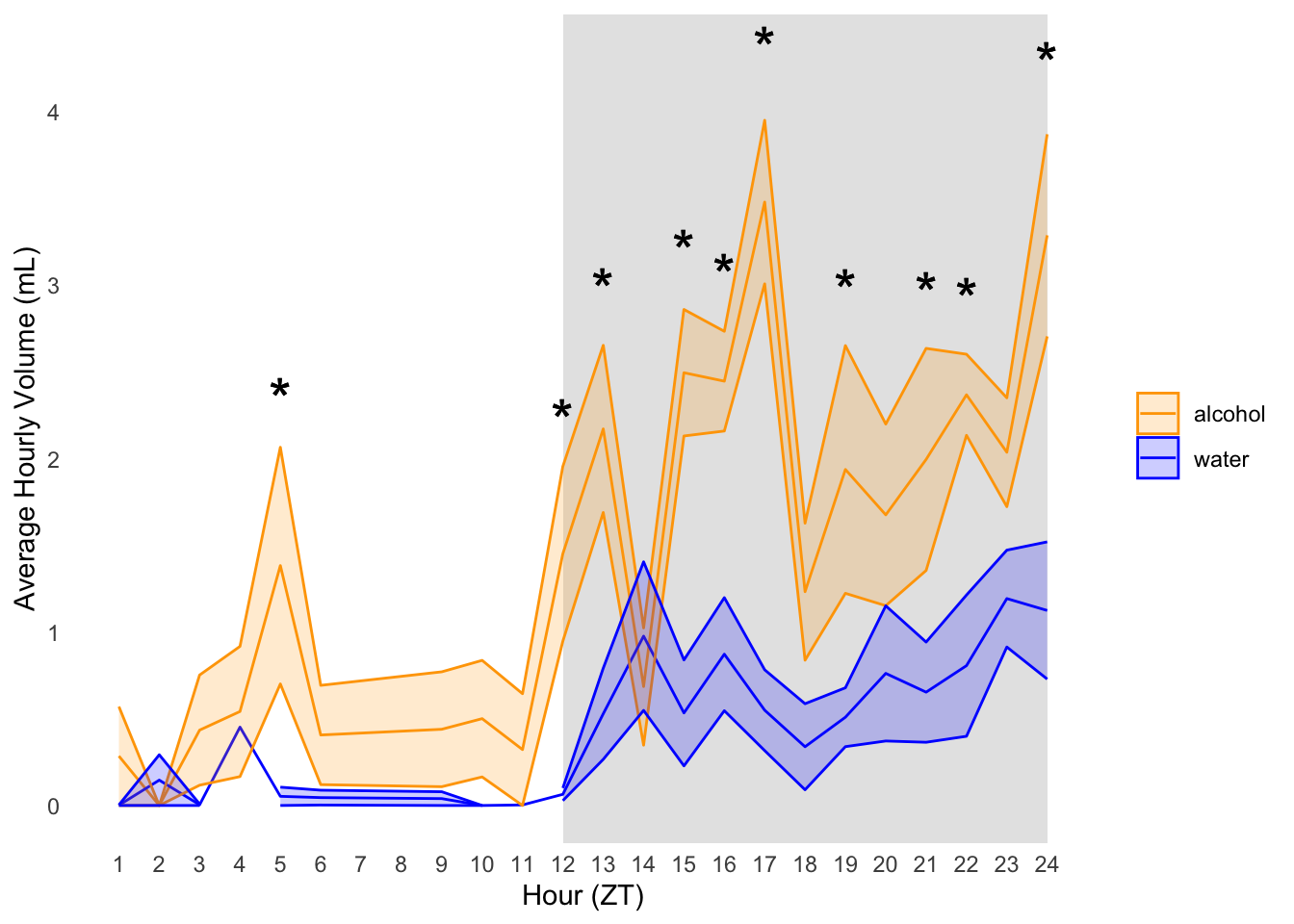


## Script 5

Graphs group alcohol preference and runs an ANOVA comparing days.

# Convert Start.Time..ms. to date format and calculate the day number
df$start_date <- as.Date(as.POSIXct(df$Start.Time..ms. / 1000, origin = "1970-01-01"))
df$day_num <- as.numeric(df$start_date - min(df$start_date)) + 1

# Summarize total volume of alcohol and water per subject per day
total_volume_by_day <- df %>%
 group_by(rat_num, day_num, fluid) %>%
 summarize(total_volume = sum(Bout.Volume..mL.), .groups = 'drop')

# Spread the data to have separate columns for alcohol and water
spread_volume_by_day <- total_volume_by_day %>%
 pivot_wider(names_from = fluid, values_from = total_volume, values_fill = list(total_volume = 0))

# Calculate Alcohol Preference
spread_volume_by_day <- spread_volume_by_day %>%
 mutate(Alcohol_Preference = ifelse(alcohol + water > 0, alcohol / (alcohol + water), 0))

# Calculate average Alcohol Preference and SEM for each day
average_preference_by_day <- spread_volume_by_day %>%
 group_by(day_num) %>%
 summarize(Avg_Alcohol_Preference = mean(Alcohol_Preference, na.rm = TRUE),
 SEM = sd(Alcohol_Preference, na.rm = TRUE) / sqrt(n()), .groups = 'drop')

# Plot the average alcohol preference with SEM error bars
ggplot(average_preference_by_day, aes(x = day_num, y = Avg_Alcohol_Preference)) +
 geom_line() +
 geom_errorbar(aes(ymin = Avg_Alcohol_Preference - SEM, ymax = Avg_Alcohol_Preference + SEM), width = 0.2) +
 scale_y_continuous(limits = c(0, 1)) +
 labs(x = "Day", y = "Average Alcohol Preference") +
 theme_minimal()


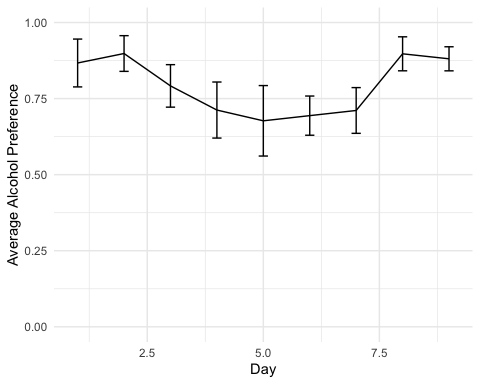


# Run an ANOVA to compare days
anova_model <- aov(Avg_Alcohol_Preference ~ factor(day_num), data = average_preference_by_day)
summary(anova_model)

## Df Sum Sq Mean Sq
## factor(day_num) 8 0.07157 0.008947

## Script 6

Graphs group total alcohol consumption and runs an ANOVA comparing days.

# Convert Start.Time..ms. to date format and calculate the day number
df$start_date <- as.Date(as.POSIXct(df$Start.Time..ms. / 1000, origin = "1970-01-01"))
df$day_num <- as.numeric(df$start_date - min(df$start_date)) + 1

# Summarize total volume of alcohol per subject per day
alcohol_volume_by_day <- df %>%
 filter(fluid == 'alcohol') %>%
 group_by(rat_num, day_num) %>%
 summarize(total_alcohol_volume = sum(Bout.Volume..mL.), .groups = 'drop')

# Calculate average total alcohol volume and SEM for each day
average_alcohol_volume_by_day <- alcohol_volume_by_day %>%
 group_by(day_num) %>%
 summarize(Avg_Alcohol_Volume = mean(total_alcohol_volume, na.rm = TRUE),
 SEM = sd(total_alcohol_volume, na.rm = TRUE) / sqrt(n()), .groups = 'drop')

# Plot the average total alcohol volume with SEM error bars
ggplot(average_alcohol_volume_by_day, aes(x = day_num, y = Avg_Alcohol_Volume)) +
 geom_line() +
 geom_errorbar(aes(ymin = Avg_Alcohol_Volume - SEM, ymax = Avg_Alcohol_Volume + SEM), width = 0.2) +
 scale_y_continuous(limits = c(0, max(average_alcohol_volume_by_day$Avg_Alcohol_Volume + average_alcohol_volume_by_day$SEM))) +
 labs(x = "Day", y = "Average Total Alcohol Volume (mL)") +
 theme_minimal()


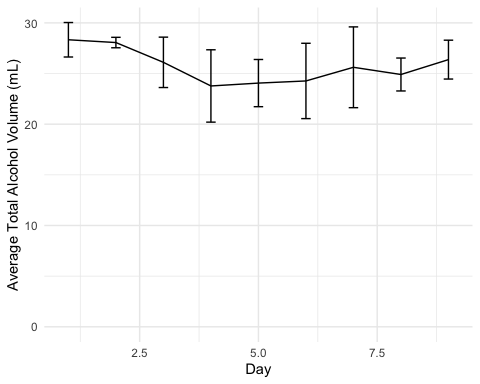


# Run an ANOVA to compare days
anova_model <- aov(Avg_Alcohol_Volume ~ factor(day_num), data = average_alcohol_volume_by_day)
summary(anova_model)

## Df Sum Sq Mean Sq
## factor(day_num) 8 22.22 2.777

## Script 7

Graphs total fluid consumption of each fluid split up by light and dark cycle and runs an anova of fluid type by time period.

# Convert Start.Time..ms. to hours and days
df$hour <- (df$Start.Time..ms. / 1000 / 3600) %% 24
df$day <- as.numeric(as.Date(as.POSIXct(df$Start.Time..ms. / 1000, origin = "1970-01-01")))

# Define light and dark periods based on the hour of the day
df$period <- ifelse(df$hour < 12, 'light', 'dark')

# Filter data for subject 1
subject1_data <- df %>%
 filter(rat_num == 1)

# Summarize total volume of alcohol and water for light and dark periods per day
volume_by_period <- subject1_data %>%
 group_by(day, period, fluid) %>%
 summarize(total_volume = sum(Bout.Volume..mL.), .groups = 'drop')

# Calculate average and SEM volume for light and dark periods
average_volume_by_period <- volume_by_period %>%
 group_by(period, fluid) %>%
 summarize(Avg_Volume = mean(total_volume),
 SEM = sd(total_volume) / sqrt(n()), .groups = 'drop')

# Plot the average volume of alcohol and water for light and dark periods
ggplot(average_volume_by_period, aes(x = fluid, y = Avg_Volume, fill = period)) +
 geom_bar(stat = "identity", position = position_dodge(), aes(fill = period)) +
 geom_errorbar(aes(ymin = Avg_Volume - SEM, ymax = Avg_Volume + SEM), width = 0.2,
 position = position_dodge(width = 0.8), width = 0.25) +
 scale_fill_manual(values = c("light" = "lightgrey", "dark" = "black")) +
 labs(x = "Fluid", y = "Average Volume (mL)", fill = "Period") +
 theme_minimal() + theme(legend.title=element_blank())

## Warning: Duplicated aesthetics after name standardisation: width


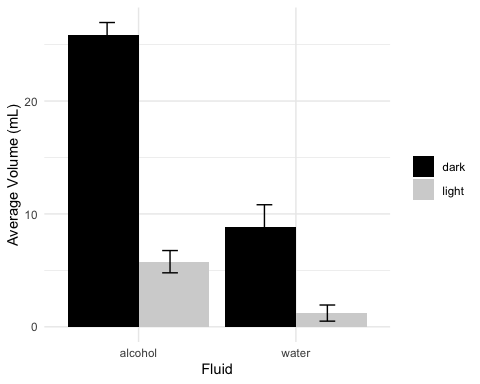


# Run an ANOVA of fluid by period
anova_model <- aov(total_volume ~ fluid * period, data = volume_by_period)
summary(anova_model)

## Df Sum Sq Mean Sq F value Pr(>F)
## fluid 1 818.1 818.1 52.21 5.9e-08 ***
## period 1 1711.5 1711.5 109.23 2.4e-11 ***
## fluid:period 1 308.5 308.5 19.69 0.000121 ***
## Residuals 29 454.4 15.7
## ---
## Signif. codes: 0 '***' 0.001 '**' 0.01 '*' 0.05 '.' 0.1 ' ' 1

## Script 8

Graphs average bout volume for bouts with at least 20 licks and runs a t-test.

# Filter events with at least 20 licks
df_filtered <- df %>%
 filter(Bout.Licks >= 20)

# Calculate average volume for each subject for events with at least 20 licks
average_volume_by_subject <- df_filtered %>%
 group_by(rat_num, fluid) %>%
 summarize(avg_bout_volume = mean(Bout.Volume..mL.), .groups = 'drop')

# Calculate the overall average and SEM of each subject's average bout volume for alcohol and water
average_volume_overall <- average_volume_by_subject %>%
 group_by(fluid) %>%
 summarize(Overall_Avg_Volume = mean(avg_bout_volume),
 SEM = sd(avg_bout_volume) / sqrt(n()), .groups = 'drop')

# Plot a bar graph with average subject bout size for alcohol and water with SEM error bars
ggplot(average_volume_overall, aes(x = fluid, y = Overall_Avg_Volume, fill = fluid)) +
 geom_bar(stat = "identity", position = position_dodge(), width = 0.7) +
 geom_errorbar(aes(ymin = Overall_Avg_Volume - SEM, ymax = Overall_Avg_Volume + SEM), width = 0.25) +
 scale_fill_manual(values = c("water" = "blue", "alcohol" = "orange")) +
 labs(x = "Fluid", y = "Average Bout Volume (mL)") +
 theme_minimal() + theme(legend.title=element_blank())


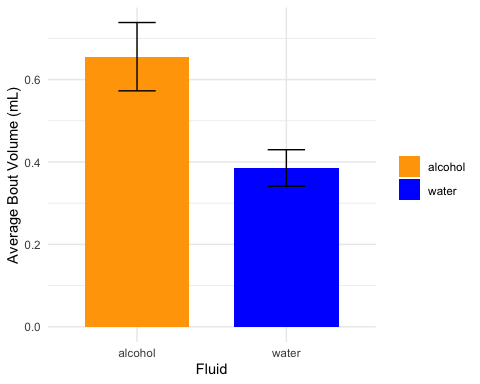


# Conduct a t-test between fluid types
t_test_result <- t.test(avg_bout_volume ~ fluid, data = average_volume_by_subject)
t_test_result

##
## Welch Two Sample t-test
##
## data: avg_bout_volume by fluid
## t = 2.8734, df = 4.5878, p-value = 0.03854
## alternative hypothesis: true difference in means between group alcohol and group water is not equal to 0
## 95 percent confidence interval:
## 0.02179305 0.51851104
## sample estimates:
## mean in group alcohol mean in group water
## 0.655564 0.385412

## Script 9

Graphs max bout volume for bouts with at least 20 licks and runs a t-test.

# Filter events with at least 20 licks
df_filtered <- df %>%
 filter(Bout.Licks >= 20)

# Calculate max volume for each subject for events with at least 20 licks
max_volume_by_subject <- df_filtered %>%
 group_by(rat_num, fluid) %>%
 summarize(max_bout_volume = max(Bout.Volume..mL.), .groups = 'drop')

# Calculate the overall average and SEM of each subject's max bout volume for alcohol and water
average_max_volume_overall <- max_volume_by_subject %>%
 group_by(fluid) %>%
 summarize(Overall_Avg_Max_Volume = mean(max_bout_volume),
 SEM = sd(max_bout_volume) / sqrt(n()), .groups = 'drop')

# Plot a bar graph with max subject bout size for alcohol and water with SEM error bars
ggplot(average_max_volume_overall, aes(x = fluid, y = Overall_Avg_Max_Volume, fill = fluid)) +
 geom_bar(stat = "identity", position = position_dodge(), width = 0.7) +
 geom_errorbar(aes(ymin = Overall_Avg_Max_Volume - SEM, ymax = Overall_Avg_Max_Volume + SEM), width = 0.25, position = position_dodge(0.7)) +
 scale_fill_manual(values = c("water" = "blue", "alcohol" = "orange")) +
 labs(x = "Fluid", y = "Max Bout Volume (mL)") +
 theme_minimal() + theme(legend.title=element_blank())


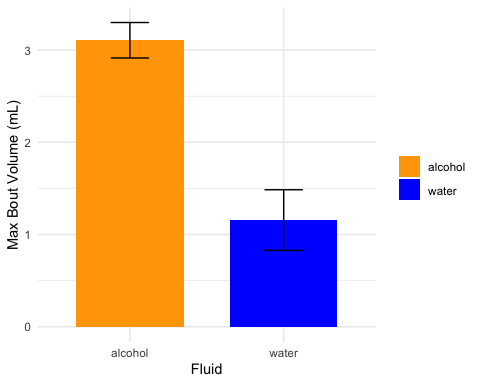


# Conduct a t-test between fluid types
t_test_result <- t.test(max_bout_volume ~ fluid, data = max_volume_by_subject)
t_test_result

##
## Welch Two Sample t-test
##
## data: max_bout_volume by fluid
## t = 5.1196, df = 4.8361, p-value = 0.004077
## alternative hypothesis: true difference in means between group alcohol and group water is not equal to 0
## 95 percent confidence interval:
## 0.9604838 2.9381162
## sample estimates:
## mean in group alcohol mean in group water
## 3.106225 1.156925

## Script 10

Graphs volume/lick for bouts with at least 20 licks and runs a t-test.

# Filter events with at least 20 licks
df_filtered <- df %>%
 filter(Bout.Licks >= 20)

# Calculate average volume per lick for each event
df_filtered <- df_filtered %>%
 mutate(volume_per_lick = Bout.Volume..mL. / Bout.Licks)

# Calculate the average volume per lick for each subject
average_volume_per_lick_by_subject <- df_filtered %>%
 group_by(rat_num, fluid) %>%
 summarize(avg_volume_per_lick = mean(volume_per_lick), .groups = 'drop')

# Calculate the overall average and SEM of each subject's average volume per lick for alcohol and water
average_volume_per_lick_overall <- average_volume_per_lick_by_subject %>%
 group_by(fluid) %>%
 summarize(Overall_Avg_Volume_Per_Lick = mean(avg_volume_per_lick),
 SEM = sd(avg_volume_per_lick) / sqrt(n()), .groups = 'drop')

# Plot a bar graph with average subject bout size for alcohol and water with SEM error bars
ggplot(average_volume_per_lick_overall, aes(x = fluid, y = Overall_Avg_Volume_Per_Lick, fill = fluid)) +
 geom_bar(stat = "identity", position = position_dodge(), width = 0.7) +
 geom_errorbar(aes(ymin = Overall_Avg_Volume_Per_Lick - SEM, ymax = Overall_Avg_Volume_Per_Lick + SEM), width = 0.25, position = position_dodge(0.7)) +
 scale_fill_manual(values = c("water" = "blue", "alcohol" = "orange")) +
 labs(x = "Fluid", y = "Average Volume Per Lick (mL/lick)") +
 theme_minimal() + theme(legend.title=element_blank())


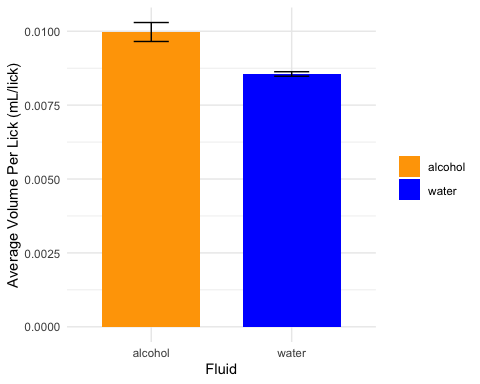


# Run a t-test comparing fluid types
t_test_result <- t.test(avg_volume_per_lick ~ fluid, data = average_volume_per_lick_by_subject)
t_test_result

##
## Welch Two Sample t-test
##
## data: avg_volume_per_lick by fluid
## t = 4.3097, df = 3.3295, p-value = 0.01856
## alternative hypothesis: true difference in means between group alcohol and group water is not equal to 0
## 95 percent confidence interval:
## 0.0004276851 0.0024118914
## sample estimates:
## mean in group alcohol mean in group water
## 0.009974039 0.008554251

## Script 11

Graphs average bout size with at least 20 licks and runs a t-test.

# Filter bouts with at least 20 licks
df_filtered <- df %>%
 filter(Bout.Licks >= 20)

# Count total number of bouts per subject for alcohol and water
bouts_count_by_subject <- df_filtered %>%
 group_by(rat_num, fluid) %>%
 summarize(total_bouts = n(), .groups = 'drop')

# Divide by total time in hours to get bouts per hour
bouts_per_hour_by_subject <- bouts_count_by_subject %>%
 mutate(bouts_per_hour = total_bouts / 240)

# Calculate the average bouts per hour for each fluid type across subjects
average_bouts_per_hour_overall <- bouts_per_hour_by_subject %>%
 group_by(fluid) %>%
 summarize(Overall_Avg_Bouts_Per_Hour = mean(bouts_per_hour),
 SEM = sd(bouts_per_hour) / sqrt(n()), .groups = 'drop')

# Plot a bar graph with average subject bouts per hour for alcohol and water with SEM error bars
ggplot(average_bouts_per_hour_overall, aes(x = fluid, y = Overall_Avg_Bouts_Per_Hour, fill = fluid)) +
 geom_bar(stat = "identity", position = position_dodge(), width = 0.7) +
 geom_errorbar(aes(ymin = Overall_Avg_Bouts_Per_Hour - SEM, ymax = Overall_Avg_Bouts_Per_Hour + SEM), width = 0.25, position = position_dodge(0.7)) +
 scale_fill_manual(values = c("water" = "blue", "alcohol" = "orange")) +
 labs(x = "Fluid", y = "Average Bouts Per Hour") +
 theme_minimal() + theme(legend.title=element_blank())


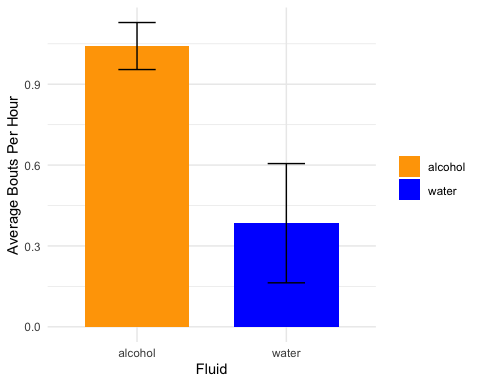


# Run a t-test comparing fluid types
t_test_result <- t.test(bouts_per_hour ~ fluid, data = bouts_per_hour_by_subject)
t_test_result

##
## Welch Two Sample t-test
##
## data: bouts_per_hour by fluid
## t = 2.764, df = 3.9113, p-value = 0.05192
## alternative hypothesis: true difference in means between group alcohol and group water is not equal to 0
## 95 percent confidence interval:
## -0.008892687 1.323476020
## sample estimates:
## mean in group alcohol mean in group water
## 1.041667 0.384375

## Script 12

Graphs average total bout volume for each fluid type split up licking bout size and runs an ANOVA and multiple comparisons tests.

# Define lick ranges
lick_ranges <- c(21, 61, 101, 141, 181, 221, 261, 301, Inf)
labels <- c('21-60', '61-100', '101-140', '141-180', '181-220', '221-260', '261-300', '300+')

# Filter out rows with lick range of 20 or less and create a factor for lick ranges
df <- df %>%
 filter(Bout.Licks >= 21) %>%
 mutate(lick_range = cut(Bout.Licks, breaks = lick_ranges, labels = labels, include.lowest = TRUE, right = FALSE))

# Calculate total volume consumed by each subject for each lick range
total_volume_by_subject <- df %>%
 group_by(rat_num, fluid, lick_range) %>%
 summarise(total_volume = sum(Bout.Volume..mL.), .groups = 'drop') %>%
 tidyr::complete(rat_num, fluid, lick_range, fill = list(total_volume = 0))

# Calculate average total volume consumed and SEM for each fluid and lick range
avg_volume_by_lick_range <- total_volume_by_subject %>%
 group_by(fluid, lick_range) %>%
 summarise(mean_volume = mean(total_volume),
 sem = sd(total_volume)/sqrt(n()), .groups = 'drop')

# ANOVA of fluid type by licking range
anova_results <- aov(total_volume ~ fluid * lick_range, data = total_volume_by_subject)
summary(anova_results)

## Df Sum Sq Mean Sq F value Pr(>F)
## fluid 1 3796 3796 35.926 2.56e-07 ***
## lick_range 7 7340 1049 9.925 1.37e-07 ***
## fluid:lick_range 7 1291 184 1.746 0.121
## Residuals 48 5071 106
## ---
## Signif. codes: 0 '***' 0.001 '**' 0.01 '*' 0.05 '.' 0.1 ' ' 1

# Pairwise comparisons of fluid at each hour
emmeans_result <- emmeans(anova_results, pairwise ~ fluid | lick_range)
pairs(emmeans_result)

## lick_range = 21-60:
## contrast estimate SE df t.ratio p.value
## alcohol - water 30.99 7.27 48 4.264 0.0001
##
## lick_range = 61-100:
## contrast estimate SE df t.ratio p.value
## alcohol - water 23.18 7.27 48 3.189 0.0025
##
## lick_range = 101-140:
## contrast estimate SE df t.ratio p.value
## alcohol - water 17.92 7.27 48 2.466 0.0173
##
## lick_range = 141-180:
## contrast estimate SE df t.ratio p.value
## alcohol - water 15.19 7.27 48 2.090 0.0419
##
## lick_range = 181-220:
## contrast estimate SE df t.ratio p.value
## alcohol - water 20.20 7.27 48 2.779 0.0078
##
## lick_range = 221-260:
## contrast estimate SE df t.ratio p.value
## alcohol - water 6.75 7.27 48 0.929 0.3574
##
## lick_range = 261-300:
## contrast estimate SE df t.ratio p.value
## alcohol - water 4.39 7.27 48 0.604 0.5487
##
## lick_range = 300+:
## contrast estimate SE df t.ratio p.value
## alcohol - water 4.60 7.27 48 0.633 0.5300

# Get the summary of pairwise comparison results
pairwise_summary <- summary(pairs(emmeans_result))

# Extract significant comparisons
significant_comparisons <- pairwise_summary[pairwise_summary$p.value < 0.05, ]
significant_lick_ranges <- significant_comparisons$lick_range

# Get max average volume for y-axis limit
max_y <- max(avg_volume_by_lick_range$mean_volume + avg_volume_by_lick_range$sem)

# Graph the average total volume consumed of each substance for each lick range
gg <- ggplot(avg_volume_by_lick_range, aes(x = lick_range, y = mean_volume, fill = fluid)) +
 geom_bar(stat = "identity", position = position_dodge(width = 0.8), width = 0.7) +
 geom_errorbar(aes(ymin = mean_volume - sem, ymax = mean_volume + sem),
 position = position_dodge(width = 0.8), width = 0.25) +
 scale_fill_manual(values = c("water" = "blue", "alcohol" = "orange")) +
 labs(x = "Lick Range", y = "Average Total Volume Consumed (mL)") +
 theme_minimal() + ylim(0, max_y*1.1) + theme(legend.title=element_blank())

# Add stars for significant pairwise comparisons
for (range in significant_lick_ranges) {
 avg_volume_range <- avg_volume_by_lick_range %>%
 filter(lick_range == range)
 if (nrow(avg_volume_range) > 0) {
 gg <- gg + geom_text(data = avg_volume_range, aes(label = "*", y = max(mean_volume+sem) *1.05), size = 8, vjust = 0, color = "black")

 }
}

# Print the plot
print(gg)


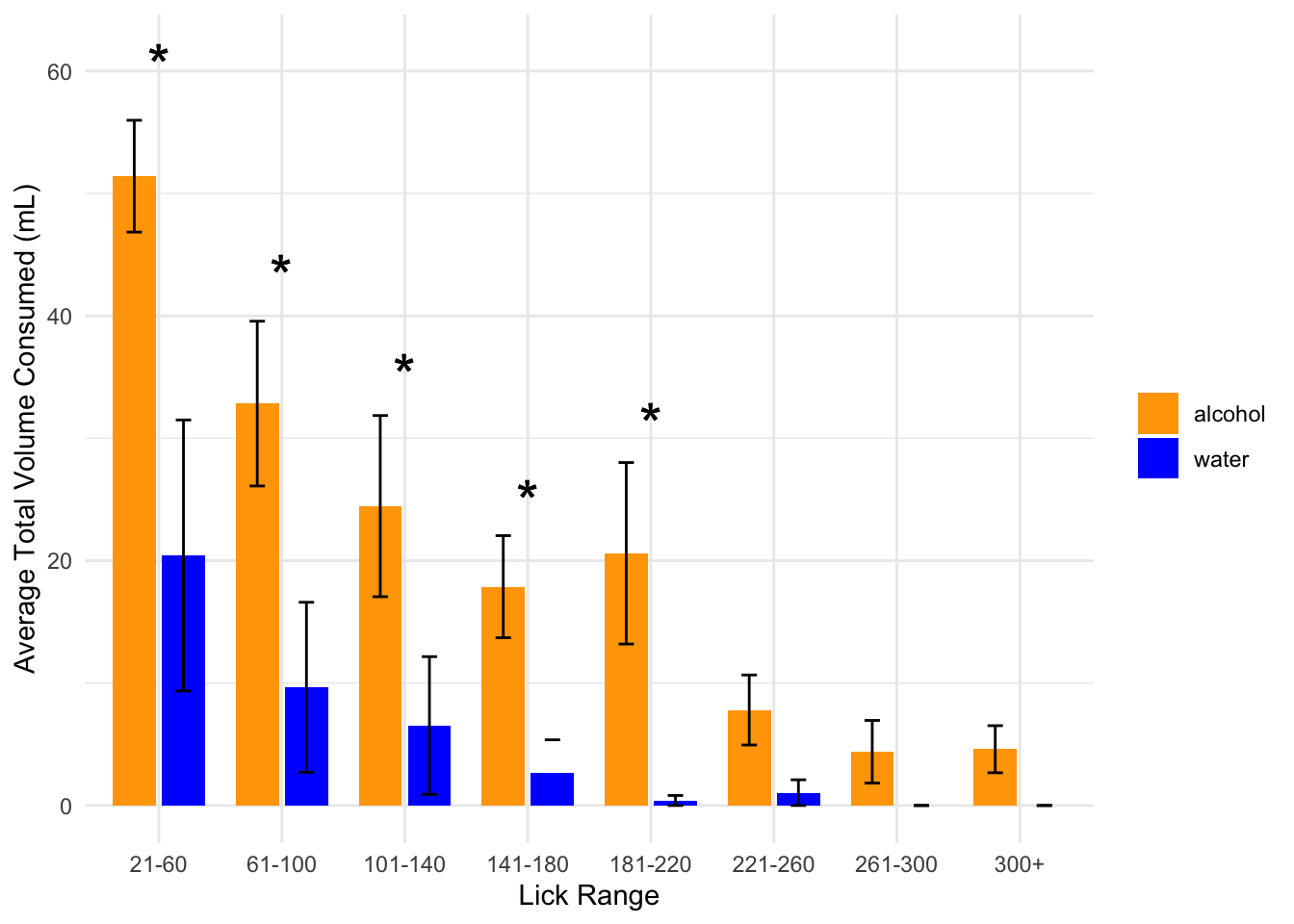


## Script 13

Graphs average total bout volume for each fluid type normalized by total volume of each fluid split up by licking bout size. Runs an ANOVA and pairwise comparisons at each lick range and plots a star above significant ranges.

# Define lick ranges
lick_ranges <- c(21, 61, 101, 141, 181, 221, 261, 301, Inf)
labels <- c('21-60', '61-100', '101-140', '141-180', '181-220', '221-260', '261-300', '300+')

# Filter out rows with lick range of 20 or less and create a factor for lick ranges
df <- df %>%
 filter(Bout.Licks >= 21) %>%
 mutate(lick_range = cut(Bout.Licks, breaks = lick_ranges, labels = labels, include.lowest = TRUE, right = FALSE))

# Calculate total volume consumed by each subject for each lick range
total_volume_by_subject <- df %>%
 group_by(rat_num, fluid, lick_range) %>%
 summarise(total_volume = sum(Bout.Volume..mL.), .groups = 'drop') %>%
 tidyr::complete(rat_num, fluid, lick_range, fill = list(total_volume = 0))

# Calculate the total volume consumed for each fluid and subject
total_volume_by_fluid_subject <- df %>%
 group_by(rat_num, fluid) %>%
 summarise(total_fluid_volume = sum(Bout.Volume..mL.), .groups = 'drop')

# Join the data frames to calculate the proportion
total_volume_proportion <- total_volume_by_subject %>%
 left_join(total_volume_by_fluid_subject, by = c("rat_num", "fluid")) %>%
 mutate(proportion = total_volume / total_fluid_volume)

# Calculate average proportion and SEM for each fluid and lick range
avg_proportion_by_lick_range <- total_volume_proportion %>%
 group_by(fluid, lick_range) %>%
 summarise(mean_proportion = mean(proportion),
 sem = sd(proportion)/sqrt(n()), .groups = 'drop')

# ANOVA of fluid type by licking range
anova_results <- aov(proportion ~ fluid * lick_range, data = total_volume_proportion)
summary(anova_results)

## Df Sum Sq Mean Sq F value Pr(>F)
## fluid 1 0.0000 0.00000 0.000 1
## lick_range 7 1.5212 0.21731 49.664 < 2e-16 ***
## fluid:lick_range 7 0.2429 0.03470 7.931 2.27e-06 ***
## Residuals 48 0.2100 0.00438
## ---
## Signif. codes: 0 '***' 0.001 '**' 0.01 '*' 0.05 '.' 0.1 ' ' 1

# Pairwise comparisons of fluid at each hour
emmeans_result <- emmeans(anova_results, pairwise ~ fluid | lick_range)
pairs(emmeans_result)

## lick_range = 21-60:
## contrast estimate SE df t.ratio p.value
## alcohol - water -0.31248 0.0468 48 -6.681 <.0001
##
## lick_range = 61-100:
## contrast estimate SE df t.ratio p.value
## alcohol - water -0.00809 0.0468 48 -0.173 0.8635
##
## lick_range = 101-140:
## contrast estimate SE df t.ratio p.value
## alcohol - water 0.03446 0.0468 48 0.737 0.4648
##
## lick_range = 141-180:
## contrast estimate SE df t.ratio p.value
## alcohol - water 0.08593 0.0468 48 1.837 0.0724
##
## lick_range = 181-220:
## contrast estimate SE df t.ratio p.value
## alcohol - water 0.11142 0.0468 48 2.382 0.0212
##
## lick_range = 221-260:
## contrast estimate SE df t.ratio p.value
## alcohol - water 0.03870 0.0468 48 0.827 0.4121
##
## lick_range = 261-300:
## contrast estimate SE df t.ratio p.value
## alcohol - water 0.02210 0.0468 48 0.472 0.6388
##
## lick_range = 300+:
## contrast estimate SE df t.ratio p.value
## alcohol - water 0.02796 0.0468 48 0.598 0.5528

# Get the summary of pairwise comparison results
pairwise_summary <- summary(pairs(emmeans_result))

# Extract significant comparisons
significant_comparisons <- pairwise_summary[pairwise_summary$p.value < 0.05, ]
significant_lick_ranges <- significant_comparisons$lick_range

# Get max average volume for y-axis limit
max_y <- max(avg_proportion_by_lick_range$mean_proportion + avg_proportion_by_lick_range$sem)

# Graph the average total volume consumed of each substance for each lick range
gg <- ggplot(avg_proportion_by_lick_range, aes(x = lick_range, y = mean_proportion, fill = fluid)) +
 geom_bar(stat = "identity", position = position_dodge(width = 0.8), width = 0.7) +
 geom_errorbar(aes(ymin = mean_proportion - sem, ymax = mean_proportion + sem),
 position = position_dodge(width = 0.8), width = 0.25) +
 scale_fill_manual(values = c("water" = "blue", "alcohol" = "orange")) +
 labs(x = "Lick Range", y = "Normalized Volume Consumed") +
 theme_minimal() + ylim(0, max_y*1.1) + theme(legend.title=element_blank())

# Add stars for significant pairwise comparisons
for (range in significant_lick_ranges) {
 avg_volume_range <- avg_proportion_by_lick_range %>%
 filter( lick_range == range)
 if (nrow(avg_volume_range) > 0) {
 gg <- gg + geom_text(data = avg_volume_range, aes(label = "*", y = max(mean_proportion+sem) *1.05), size = 8, vjust = 0, color = "black")

 }
}

# Print the plot
print(gg)


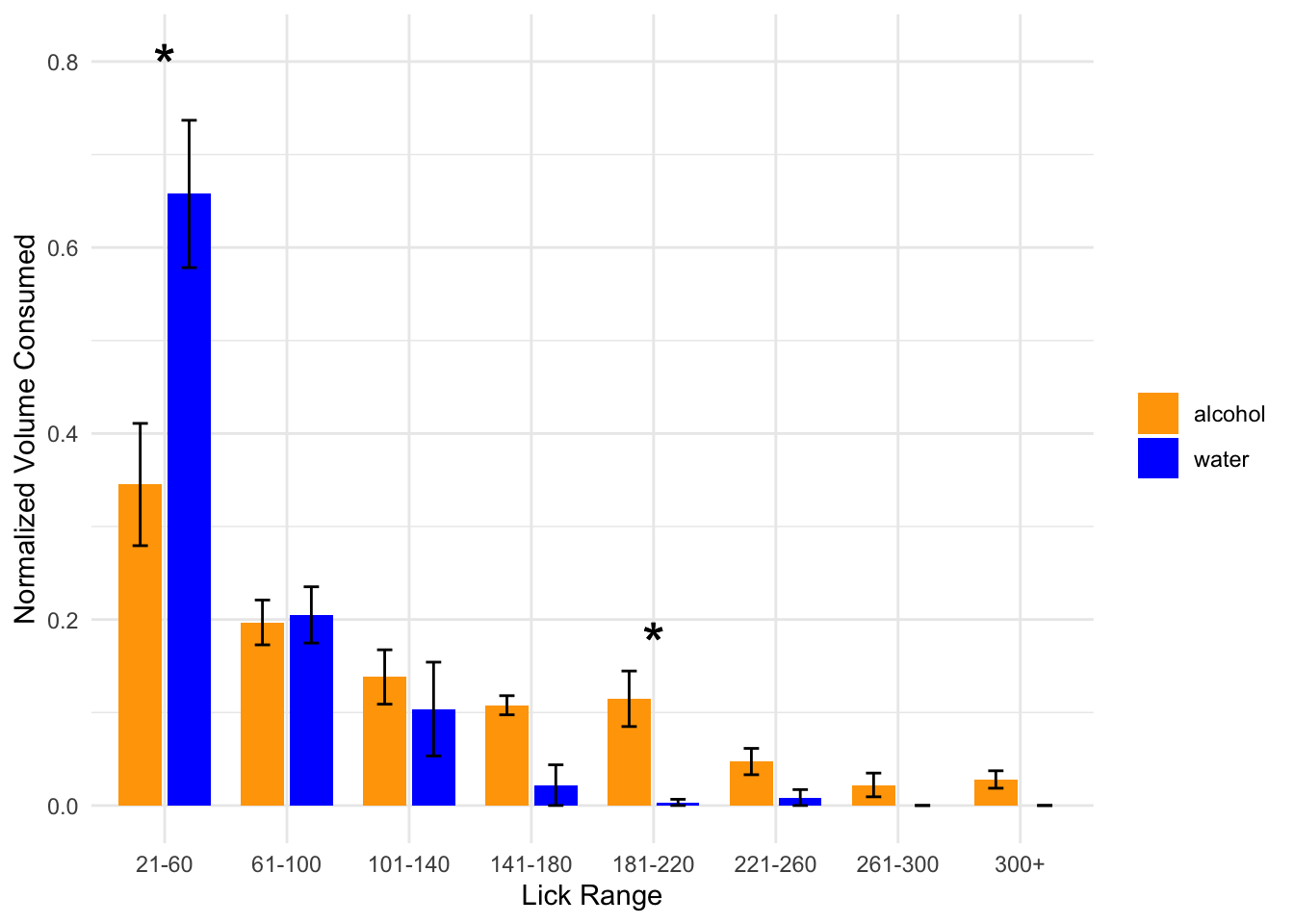

Supplement: Supplementary file 1 — Supplementary Materials [file 44277_2024_2_MOESM1_ESM.docx]
